# *Calotropis gigantea* stem bark extracts inhibit liver cancer induced by diethylnitrosamine

**DOI:** 10.1038/s41598-022-16321-0

**Published:** 2022-07-15

**Authors:** Suphunwadee Sawong, Dumrongsak Pekthong, Pennapha Suknoppakit, Thanwarat Winitchaikul, Worasak Kaewkong, Julintorn Somran, Chaidan Intapa, Supawadee Parhira, Piyarat Srisawang

**Affiliations:** 1 Department of Physiology, Faculty of Medical Science, Naresuan University, Phitsanulok, 65000 Thailand; 2 Department of Pharmacy Practice, Faculty of Pharmaceutical Sciences, Naresuan University, Phitsanulok, 65000 Thailand; 3 Department of Biochemistry, Faculty of Medical Science, Naresuan University, Phitsanulok, 65000 Thailand; 4 Department of Pathology, Faculty of Medicine, Naresuan University, Phitsanulok, 65000 Thailand; 5 Department of Oral Diagnosis, Faculty of Dentistry, Naresuan University, Phitsanulok, 65000 Thailand; 6 Department of Pharmaceutical Technology, Faculty of Pharmaceutical Sciences, Naresuan University, Phitsanulok, 65000 Thailand

**Keywords:** Cancer, Drug discovery, Diseases

## Abstract

Several fractions of *Calotropis gigantea* extracts have been proposed to have potential anticancer activity in many cancer models. The present study evaluated the anticancer activity of *C. gigantea* stem bark extracts in liver cancer HepG2 cells and diethylnitrosamine (DEN)-induced primary liver cancer in rats. The carcinogenesis model induced by DEN administration has been widely used to study pathophysiological features and responses in rats that are comparable to those seen in cancer patients. The dichloromethane (CGDCM), ethyl acetate, and water fractions obtained from partitioning crude ethanolic extract were quantitatively analyzed for several groups of secondary metabolites and calactin contents. A combination of *C. gigantea* stem bark extracts with doxorubicin (DOX) was assessed in this study to demonstrate the enhanced cytotoxic effect to cancer compared to the single administration. The combination of DOX and CGDCM, which had the most potential cytotoxic effect in HepG2 cells when compared to the other three fractions, significantly increased cytotoxicity through the apoptotic effect with increased caspase-3 expression. This combination treatment also reduced ATP levels, implying a correlation between ATP and apoptosis induction. In a rat model of DEN-induced liver cancer, treatment with DOX, *C. gigantea* at low (CGDCM-L) and high (CGDCM-H) doses, and DOX + CGDCM-H for 4 weeks decreased the progression of liver cancer by lowering the liver weight/body weight ratio and the occurrence of liver hyperplastic nodules, fibrosis, and proliferative cells. The therapeutic applications lowered TNF-α, IL-6, TGF-β, and α-SMA inflammatory cytokines in a similar way, implying that CGDCM had a curative effect against the inflammation-induced liver carcinogenesis produced by DEN exposure. Furthermore, CGDCM and DOX therapy decreased ATP and fatty acid synthesis in rat liver cancer, which was correlated with apoptosis inhibition. CGDCM reduced cleaved caspase-3 expression in liver cancer rats when used alone or in combination with DOX, implying that apoptosis-inducing hepatic carcinogenesis was suppressed. Our results also verified the low toxicity of CGDCM injection on the internal organs of rats. Thus, this research clearly demonstrated a promising, novel anticancer approach that could be applied in future clinical studies of CGDCM and combination therapy.

## Introduction

Liver cancer is one of the most aggressive occurrences of cancer with a high fatality rate worldwide^1^. Hepatocellular carcinoma (HCC) is the most common type of primary liver cancer, with cholangiocarcinoma accounting for the remainder^2^. However, following standard chemotherapy with or without surgical resection, a poor prognosis is still established. Therapeutic regimens using traditional plant extracts are being examined as an alternative therapeutic strategy to overcome the limitations of current treatments. Although animal models for studying tumor progression are readily available and convenient for exploring potential therapeutics, diethylnitrosamine (DEN)-induced hepatocarcinogenesis serves as a primary liver cancer model that illustrates the mechanisms of inflammatory responses, cirrhosis, and carcinogenesis processes^3–5^. Numerous studies have shown that plant extracts have anticancer properties by suppressing DEN-induced liver cancer. DEN-induced hepatocellular carcinogenesis in rats was shown to be reduced by the ethanolic extract of *Solanum xanthocarpum* Schrad. & Wendl leaves, which also exhibited an antioxidant effect^6^. This report is consistent with the antioxidant activity of extracts from *Punica granatum* (pomegranate) peel and seed oil^7^, blueberries^8^, lotus *Nelumbo nucifera* leaves^9^, ajwa dates (*Phoenix dactylifera* L.)^10^, and *Solanum trilobatum*^11^, which inhibited DEN-induced hepatic injury in animal models. Ganoderic acid A isolated from *Ganoderma lucidum*^12^; triterpenoid compounds found in the aerial parts of *Wedelia calendulacea*^13^, and dihydrochalcone flavonoids present in the leaves, bark, and fruit of apple trees decreased oxidative stress and inflammatory responses^14^, which could inhibit carcinogenesis against DEN exposure. Altogether, compounds in the abovementioned plant extracts have been proposed to possess potential anticancer activity.

*Calotropis gigantea* (Apocynaceae, Asclepedaceae) is commonly used globally as a traditional medicine for the treatment of several illnesses. This plant is widely grown in many countries in Africa, Eastern Asia, and Southeast Asia, including Thailand. Phytochemical components isolated from this plant include cardiac glycosides^15^, phenolics^16^, triterpenoids^17^, flavonoids^18^, alkaloids, etc. Extracts from all parts of this plant have been shown to have a variety of biological activities^19–23^. However, the preventive and anticancer effects have received much attention, but the underlying mechanism of these effects is poorly understood. Recently, our continuing experiment of *C. gigantea* stem bark extracts has become interesting as a potential treatment for cancer cells by mediating the apoptosis mechanism. However, more study in additional cancer models is required to establish its intriguing therapeutic potential for further applications.

The present study addressed the anticancer activity of extracts from stem bark of *C. gigantea*, which were crude extracts collected from 95% ethanol (CGEtOH) to obtain dichloromethane (CGDCM), ethyl acetate (CGEtOAc), and water (CGW) fractions. The suppression of adenosine triphosphate (ATP) production, which mediates apoptotic activity, was investigated in HepG2 cells in this study. The extracts, when combined with the lowest dose of doxorubicin (DOX), the anthracycline antibiotic that inhibits topoisomerase II, were hypothesized to improve the anticancer activity of the extract compared to monotherapy, with less adverse toxicity to the heart, liver, kidneys, and testis, as previously reported^14,24–26^. In addition, HCC development in rats was studied using DEN to evaluate the effect of the extracts from stem bark of *C. gigantea* and a combination with DOX on the suppression of hepatocarcinogenesis over the course of 4 weeks of treatment.

The findings of this research may provide valuable information for the development of low-risk cancer treatments based on plant extracts. The combined therapeutic advantages of *C. gigantea* stem bark extracts may be beneficial for future anticancer treatment regimens.

## Results

### Extraction and phytochemical determination of the *C. gigantea* stem bark extracts

**Figure 1:**
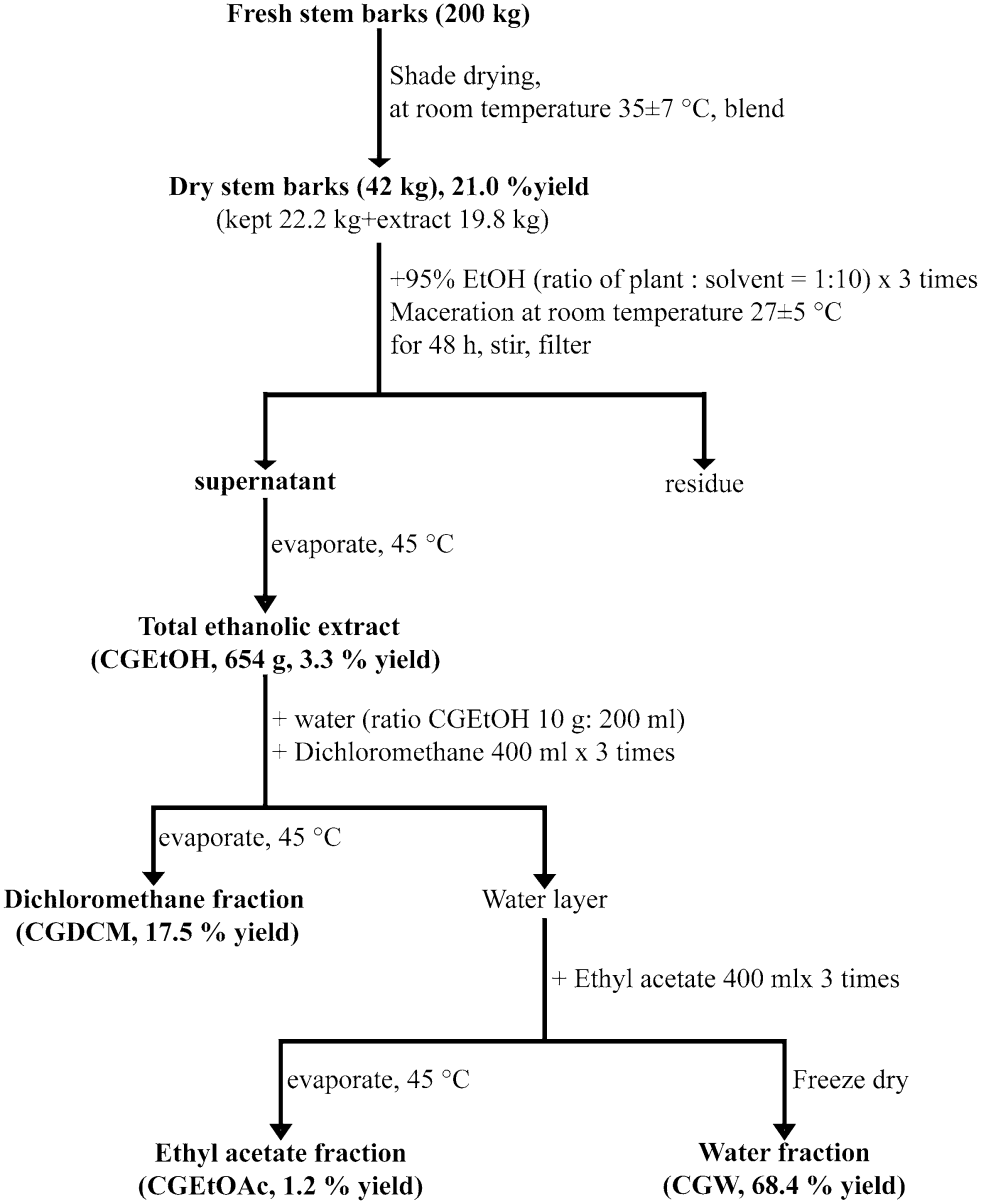
Extraction and fractionation protocols of *C. gigantea* stem bark to obtain the total ethanolic extract (CGEtOH), dichloromethane fraction (CGDCM), ethyl acetate fraction (CGEtOAc) and water fraction (CGW).

Figure 1 shows the extraction and fractionation protocols of the stem bark of *C. gigantea*. The results indicated that the percent yield of dry *C. gigantea* stem bark was 21.0% of that of fresh stem bark. The 19.8 kg dry stem bark samples were extracted with 95% ethanol to obtain the total ethanolic extract (CGEtOH, 654 g, 3.3% of dry stem bark). CGEtOH (300 g) was subjected to liquid–liquid partition to obtain the dichloromethane fraction (CGDCM, 52.5 g, 17.5% yield of CGEtOH), ethyl acetate fraction (CGEtOAc, 3.7 g, 1.2% yield), and water fraction (CGW, 205.3 g, 68.4% yield). The CGEtOH extract, along with its fractions, CGDCM, CGEtOAc, and CGW, were further analyzed to determine the contents of several groups of secondary metabolites and calactin, one of the major cardenolides with anticancer activity, and their in vitro and in vivo bioactivities.

#### Total cardiac glycoside content

**Figure 2:**
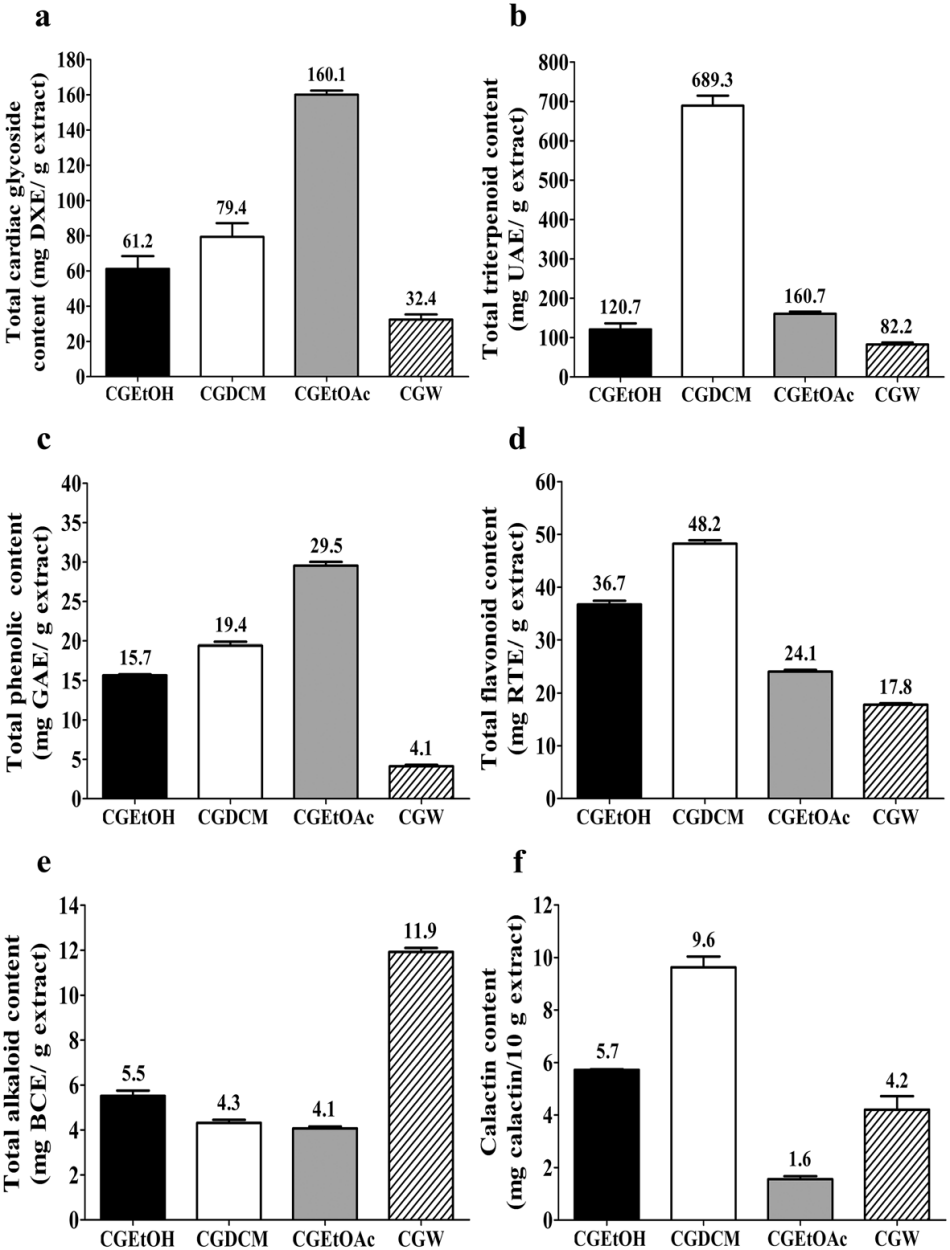
Phytochemical contents of *C. gigiantea* stem bark extracts, (**a**) total cardiac glycoside content (mg DXE/g extract), (**b**) total triterpenoid content (mg UAE/g extract), (**c**) total phenolic content (mg GAE/g extract), (**d**) total flavonoid content (mg RTE/g extract), (**e**) total alkaloid content (mg BCE/g extract) and (**d**) Calactin content (mg calactin/10 g extract), where DXE is digoxin equivalent, UAE is ursolic acid equivalent, GAE is gallic acid equivalent, RTE is rutin equivalent and BCE is berberine chloride equivalent. CGEtOH, CGDCM, CGEtOAc, and CGW are the total ethanolic extract, dichloromethane fraction, ethyl acetate fraction and water fraction of *C. gigantea* stem bark, respectively.

Figure 2a illustrates the total cardiac glycoside contents of all tested samples. It was found that CGEtOAc contained the highest amount of cardiac glycosides (160.1 ± 2.33 mg DXE/g extract), as expected, while CGDCM and CGEtOH had similar cardiac glycoside contents of 79.4 ± 7.74 and 61.2 ± 7.27 mg DXE/g extract, respectively. The lowest cardiac glycoside content was observed in CGW (32.4 ± 2.93 mg DXE/g extract). All of the tested extracts contained high amounts of cardiac glycosides in the range of 32.4–160.1 mg DXE/g extract.

#### Total triterpenoid content

CGDCM was the fraction that contained the highest amount of triterpenoids (689.3 ± 25.59 mg UAE/g extract), likely owing to its nonpolar nature^27,28^. The total triterpenoid content of CGEtOAc (160.7 ± 5.25 mg UAE/g extract) was higher than that of CGEtOH (120.7 ± 15.57 mg UAE/g extract), as shown in Fig. 2b. The lowest amounts of triterpenoids were observed in CGW (82.2 ± 5.09 mg UAE/g extract), as expected due to the polarity of water.

#### Total phenolic content

The extracts from *C. gigantea* stem bark consisted of phenolic compounds in the range of 4.1–29.5 mg GAE/g extract. CGEtOAc exhibited the highest total phenolic content, followed by CGDCM, CGEtOH, and CGW, with contents of approximately 29.5 ± 0.79, 19.4 ± 0.48, 15.7 ± 0.13 and 4.1 ± 0.19 mg GAE/g extract, respectively, as shown in Fig. 2c.

#### Total flavonoid content

A summary of the total flavonoid contents of the tested *C. gigantea* extracts is illustrated in Fig. 2d. The results indicated that CGDCM contained the highest flavonoid content (48.2 ± 0.64 mg RTE/g extract), while the total flavonoid contents of CGEtOH, CGEtOAc, and CGW were 36.7 ± 0.68, 24.1 ± 0.33 and 17.8 ± 0.30 mg RTE/g extract, respectively.

#### Total alkaloid content

Several parts of *C. gigantea* have been reported to contain some alkaloids^29^. The results indicated that CGW contained the highest amount of alkaloids, followed by CGEtOH, CGDCM, and CGEtOAc, which showed similar contents, with values of approximately 11.9 ± 0.18, 5.5 ± 0.24, 4.3 ± 0.14 and 4.1 ± 0.08 mg BCE/g extract, respectively, as presented in Fig. 2e.

#### Calactin content

**Figure 3:**
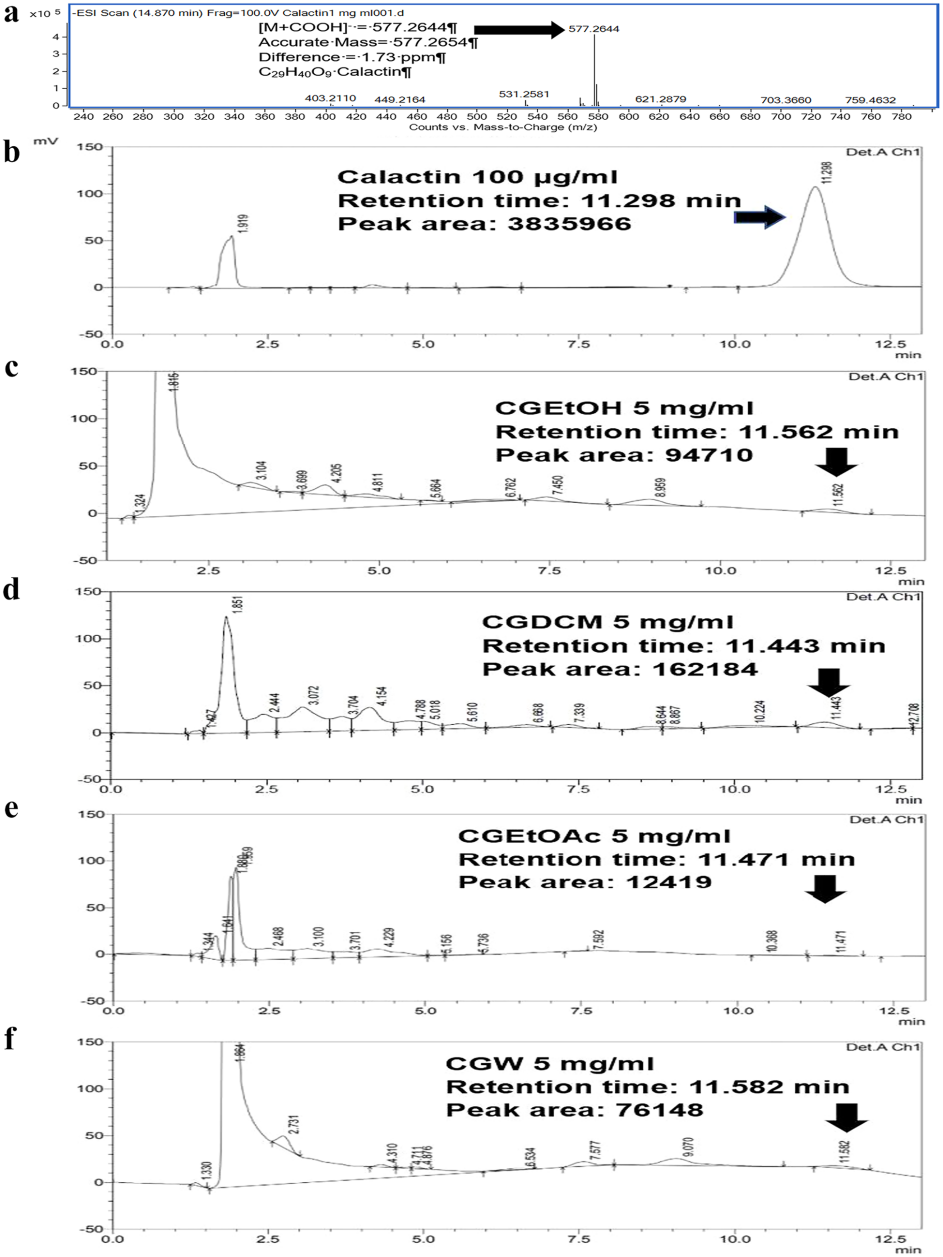
HPLC analysis: mass spectra of calactin (**a**), HPLC chromatograms of calactin (**b**), CGEtOH (**c**), CGDCM (**d**), CGEtOAc (**e**) and CGW (**f**), which are the total ethanolic extract, dichloromethane fraction, ethyl acetate fraction and water fraction of *C. gigantea* stem bark, respectively.

Calactin is a promising anticancer cardenolide found in *C. gigantea*^30^. This compound was used to quantify and represent an active compound in the extracts from *C. gigantea* in this study. Figure 3a,b illustrates the mass spectra and HPLC chromatogram of standard calactin, respectively, showing a calactin peak at a retention time of approximately 11.43 ± 0.16 min. High-resolution mass spectrometry was used to confirm the molecular weight of the analyte, as shown in Fig. 3a (found *m/z* of [M+HCOO]^−^ = 577.2644, calculated *m/z* of [M+HCOO]^−^ = 577.2654, accurate mass = 532.2672 (C_29_H_40_O_9_), difference of approximately 1.73 ppm). The calactin contents of the tested extracts were investigated according to the Kharat method^31^. The HPLC chromatograms of CGEtOH, CGDCM, CGEtOAc, and CGW are displayed in Fig. 3c–f. Several peaks appeared in the chromatograms. The peak area at the retention time approximately 11.43 ± 0.16 min attributed to calactin was used to calculate the amount of calactin in each sample. A summary of the calactin content is shown in Fig. 2f. It was found that CGDCM contained the highest amount of calactin, followed by CGEtOH, CGW, and CGEtOAc, with values of approximately 9.6 ± 0.41, 5.7 ± 0.03, 4.2 ± 0.51 and 1.6 ± 0.11 mg calactin/10 g extract, respectively.

### Cell cytotoxic activity of *C. gigantea* extracts in HepG2 cells

**Figure 4:**
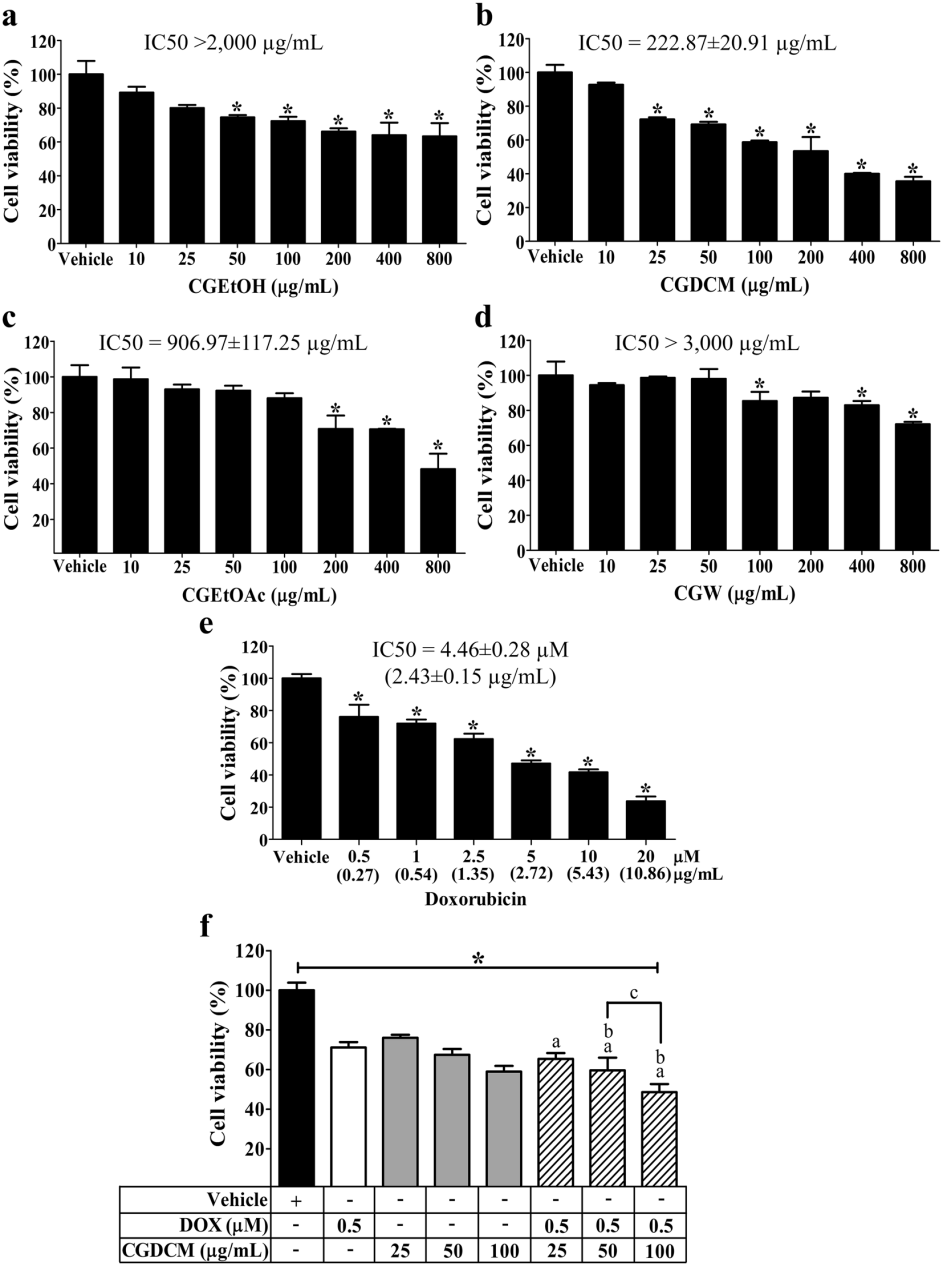
Cytotoxicity of *C. gigantea* stem bark extracts on cell viability of HepG2 cells in 24 h. Analysis was performed by MTT. The viabilities of cells after treatment with the CGEtOH, CGDCM, CGEtOAc, and CGW fractions are shown in (**a**–**d**). The vehicle was 0.8% DMSO treatment. (**e**) DOX effect, (**f**) and the combination of DOX with CGDCM on HepG2 cell cytotoxicity. The data were analyzed using one-way ANOVA with Tukey’s HSD test and reported as the mean ± SD of three independent experiments (n = 3). *p < 0.05 compared to the vehicle group, a; p < 0.05 compared to the doxorubicin group, b; p < 0.05 compared to the treatment with CGDCM extract alone group. CGEtOH, *C. gigantea* ethanolic extract; CGDCM, *C. gigantea* dichloromethane extract; CGEtOAc, *C. gigantea* ethyl acetate extract; CGW, *C. gigantea* water extract; DOX, doxorubicin.

The toxicity of four fractions of extracts from *C. gigantea* stem bark, CGEtOH, CGDCM, CGEtOAc, and CGW, on HepG2 cells was initially determined using the MTT test. As shown in Fig. 4a–d, cells incubated for 24 h were significantly inhibited with IC_50_ values of > 2000 µg/mL, 222.87 ± 20.91 µg/mL, 906.97 ± 117.25 µg/mL, and > 3000 µg/mL for CGEtOH, CGDCM, CGEtOAc, and CGW, respectively. CGDCM was shown to be the most effective at reducing HepG2 cell viability. The IC_50_ of DOX for HepG2 cells was 4.46 ± 0.28 µM (2.43 ± 0.15 µg/mL) (Fig. 4e). From these results, sub-IC_50_ values of DOX at 0.5 µM (0.27 μg/mL) and CGDCM at 25, 50, and 100 µg/mL were chosen for further combination therapy experiments based on these findings.

The combinations of 0.5 µM DOX (0.27 μg/mL) with 50 or 100 µg/mL CGDCM significantly promoted cell death after 24 h of incubation when compared to the vehicle and DOX or CGDCM treatment alone, but 0.5 µM DOX (0.27 μg/mL) combined with 100 µg/mL CGDCM had the most potent cytotoxic impact in HepG2 cells (Fig. 4f). Thus, apoptotic induction experiments were then carried out using combinations of 0.5 µM DOX (0.27 μg/mL) with CGDCM (25, 50 and 100 µg/mL).

### Combinations of DOX and CGDCM induced apoptosis related to ATP production and inhibited invasive activity in HepG2 cells

**Figure 5:**
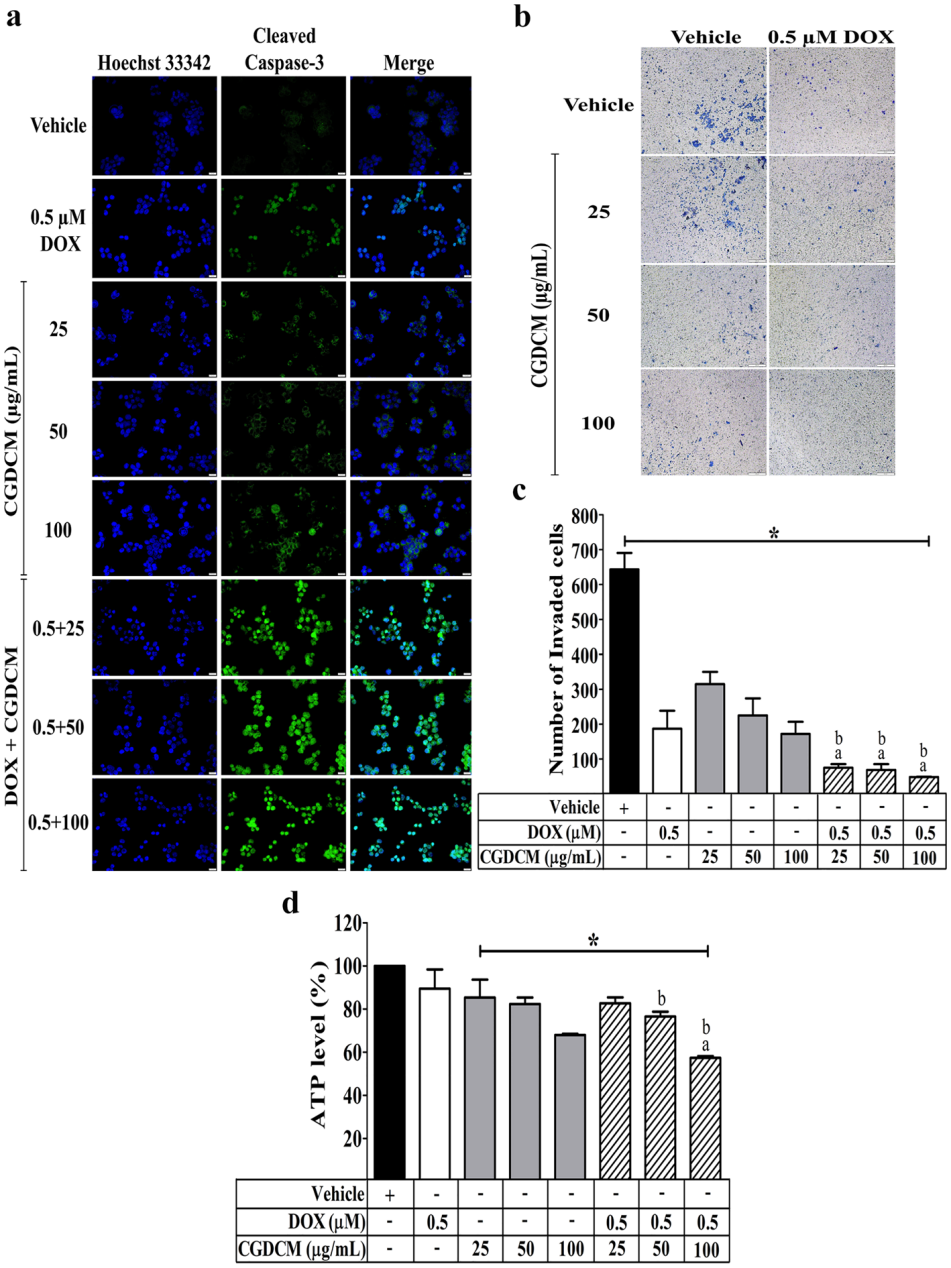
The effect of CGDCM extract from stem bark of *C. gigantea* in the induction of apoptosis and in the inhibition of invasive activity in HepG2 cells treated with CGDCM (25, 50, and 100 µg/mL), DOX (0.5 μM), and combinations of the two for 24 h. (**a**) Representative images of apoptosis evaluated by the expression of cleaved caspase-3 with counterstained nuclei with Hoechst 33342 and visualized by fluorescence microscopy, bars = 20 µm. (**b**) Representative images of the anti-invasion effect evaluated by a Transwell assay and visualized under a light microscope, bars = 200 µm, and (**c**) histogram showing the total number of invading cells. (**d**) The production of ATP was expressed as a percentage compared with the vehicle control. Vehicle cells were treated with 0.8% DMSO. The data were analyzed using one-way ANOVA with Tukey’s HSD test and reported as the mean ± SD of three independent experiments (n = 3). *p < 0.05 compared to the vehicle group, a; p < 0.05 compared to the doxorubicin group, b; p < 0.05 compared to the treatment with CGDCM extract alone group. CGDCM, *C. gigantea* dichloromethane extract; DOX, doxorubicin.

The capacity of DOX, CGDCM, and a combination of the two to cause apoptosis in HepG2 cells was investigated. Figure 5a shows that combining 0.5 µM DOX (0.27 μg/mL) with CGDCM (25, 50 and 100 µg/mL) enhanced apoptosis in HepG2 cells over 24 h, as seen by increased cleaved caspase-3 expression.

The invasive ability of cancer cells was next investigated using 0.5 µM DOX (0.27 μg/mL) in combination with CGDCM (25, 50 and 100 µg/mL). The combination treatment significantly reduced the invading cells and the relative invasive capabilities (%) of the cells when compared to the treatment of either drug alone at the corresponding concentration (Fig. 5b,c).

ATP has been reported to contribute to the apoptotic induction activity of many anticancer agents in several cancer cells^32–38^. As a result, we investigated the mechanism of apoptotic induction mediation by reducing ATP levels after 24 h of treatment with DOX and CGDCM. We found that combining 0.5 µM DOX (0.27 μg/mL) with 100 µg/mL CGDCM resulted in lower ATP levels than either treatment alone at the corresponding concentration (Fig. 5d). Thus, this combination of low concentrations of DOX and CGDCM exhibited apoptosis in HepG2 cells more effectively than either agent alone by targeting the suppression of ATP production.

### Combined DOX and CGDCM treatment reduced liver injury in DEN-induced HCC rats

**Figure 6:**
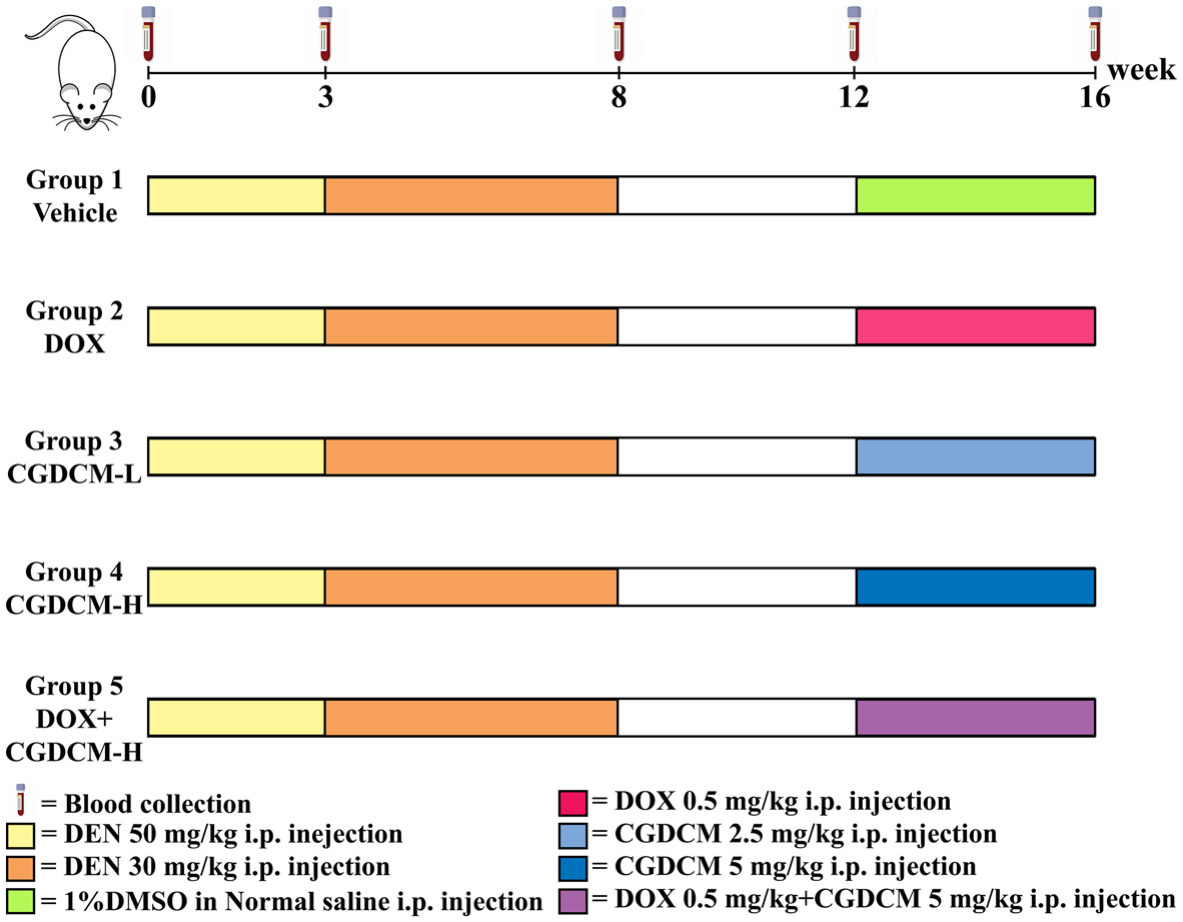
Experimental design of DEN-induced liver cancer rats. DEN administration was IP injected at a dose of 50 mg/kg twice a week for 3 weeks and then injected with a dose of 30 mg/kg twice a week for another 5 weeks until week 8. Then, DEN injection was stopped for 4 weeks (week 8 to week 12). The treatments were performed for 4 weeks from week 12. Randomization groups included DOX, CGDCM-L, CGDCM-H, and the combination of DOX with CGDCM-H. The vehicle group was treated with 0.8% DMSO. CGDCM, *C. gigantea* dichloromethane extract; CGDCM-L, CGDCM low dose; CGDCM-H, CGDCM high dose; DOX, doxorubicin.

Based on an in vitro IC_50_ investigation, the doses of CGDCM used in animal treatments were established at approximately 100 times the IC_50_ value^22,39,40^. *C. gigantea* extracts at doses ranging from 2 to 10 mg/kg have been reported to exhibit anticancer activity in animal models^17,41^. Thus, CGDCM-L and CGDCM-H doses of 2.5 mg/kg and 5 mg/kg, respectively, were employed for therapy in DEN-induced HCC rats, as shown in the experimental design in Fig. 6.

**Figure 7:**
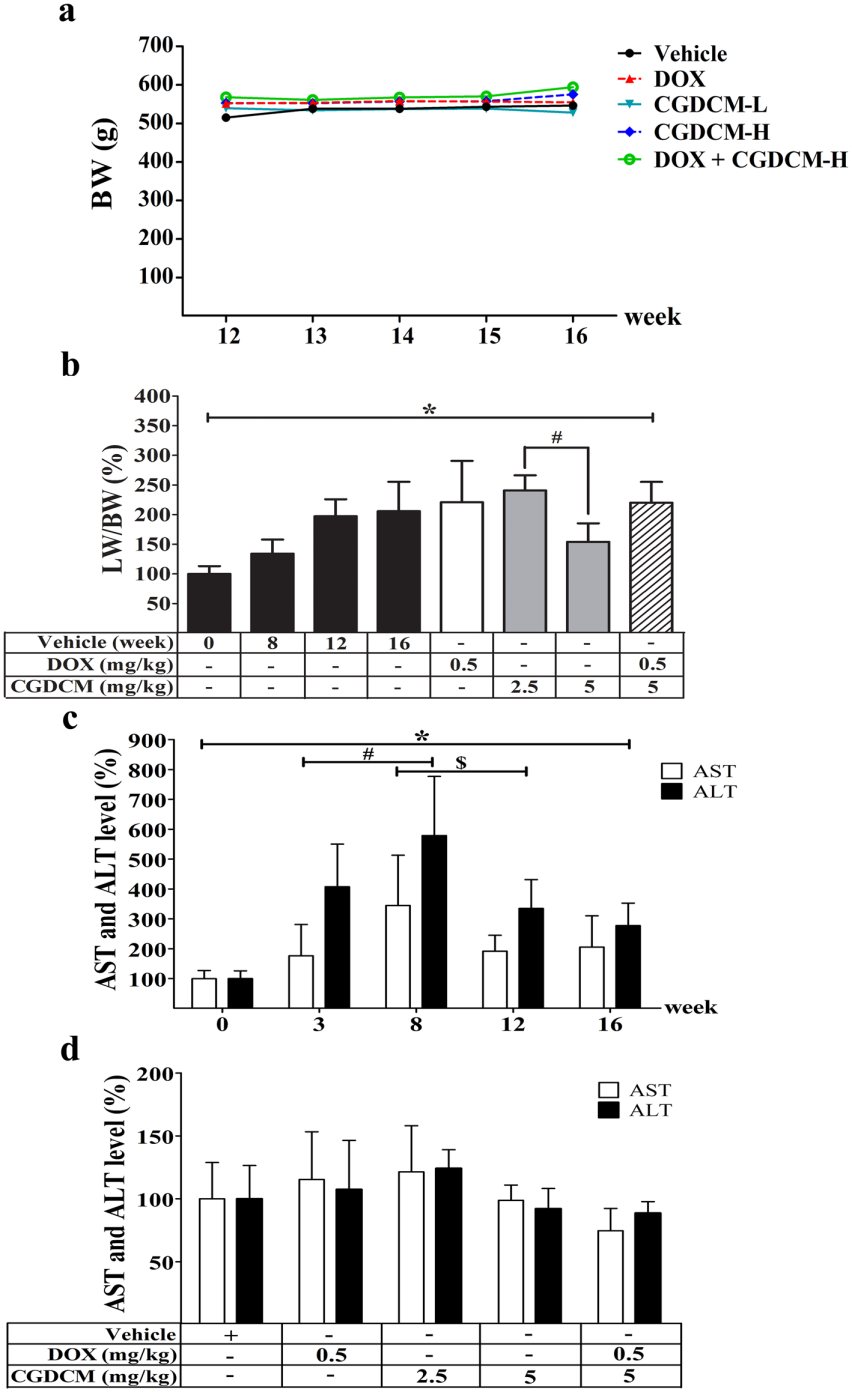
Effect of CGDCM extract from stem bark of *C. gigantea* on DEN-induced liver cancer rats. DEN administration was performed for 8 weeks, and the treatments were performed for 4 weeks from week 12, which included DOX, CGDCM-L, CGDCM-H, and the combination of DOX and CGDCM-H. The vehicle group was treated with 0.8% DMSO. (**a**) The body weight of the rats was measured weekly until week 16. (**b**) A percentage of the liver-to-body weight ratio was recorded at the end of the treatments (week 16). (**c**) A percentage of serum levels of AST and ALT were analyzed in weeks 0, 3, 8, 12 and 16 and (**d**) at week 16 of the treatment groups. The data were analyzed using one-way ANOVA with Tukey’s HSD test and reported as the mean ± SD (n = 7), *p < 0.05 compared to the vehicle at week 0. CGDCM*, C. gigantea* dichloromethane extract; CGDCM-L, CGDCM low dose; CGDCM-H, CGDCM high dose; DOX*,* doxorubicin.

DEN had no effect on the body weight of rats in this experiment after the DEN injection was stopped at week 8 and evidence cancer was allowed to grow for another 4 weeks. The body weight of the rats remained constant across all groups from week 12 to week 16 of the experiment. When rats were given DOX or CGDCM, their body weight did not change when compared to that of rats in the vehicle control group (Fig. 7a). Rats exposed to DEN experienced a significant increase in the relative liver-to-body weight ratio from week 12 to week 16. When compared to the vehicle control group, therapy with CGDCM-H for 4 weeks dramatically reduced the relative liver-to-body weight ratio (Fig. 7b). The combination of 0.5 mg/kg DOX and CGDCM-H did not lower the rat liver-to-body weight ratio as much as using CGDCM-H alone.

As hepatic functional indices, aspartate aminotransferase (AST) and alanine aminotransferase (ALT) levels are important markers of hepatic injury. After hepatocyte rupture, these enzymes are released into circulation^42–44^. Following DEN administration, there was a significant increase in AST and ALT levels, which was highest at week 8. When the DEN injection was stopped at week 8, the levels of AST and ALT were greatly reduced at weeks 12 and 16, but they remained higher than those seen in the control group before injection at week 0 (Fig. 7c). The rats given DOX, CGDCM-L, CGDCM-H, or a combination of DOX and CGDCM-H for 4 weeks (until week 16) exhibited no significant reduction in AST and AST levels when compared to rats in the vehicle control group. However, the levels of these two parameters tended to be low in the CGDCM-H and DOX combined with CGDCM-H groups (Fig. 7d).

### In DEN-induced HCC rats, a combination of DOX and CGDCM reduced liver nodules, fibrosis, and carcinogenesis

#### Macroscopic examination

**Figure 8:**
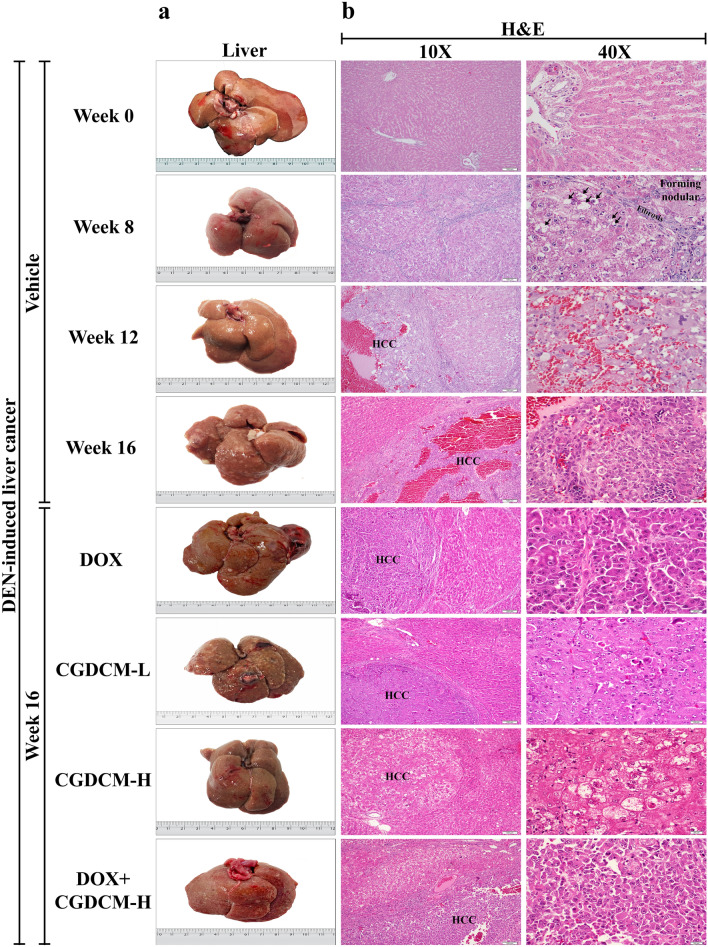

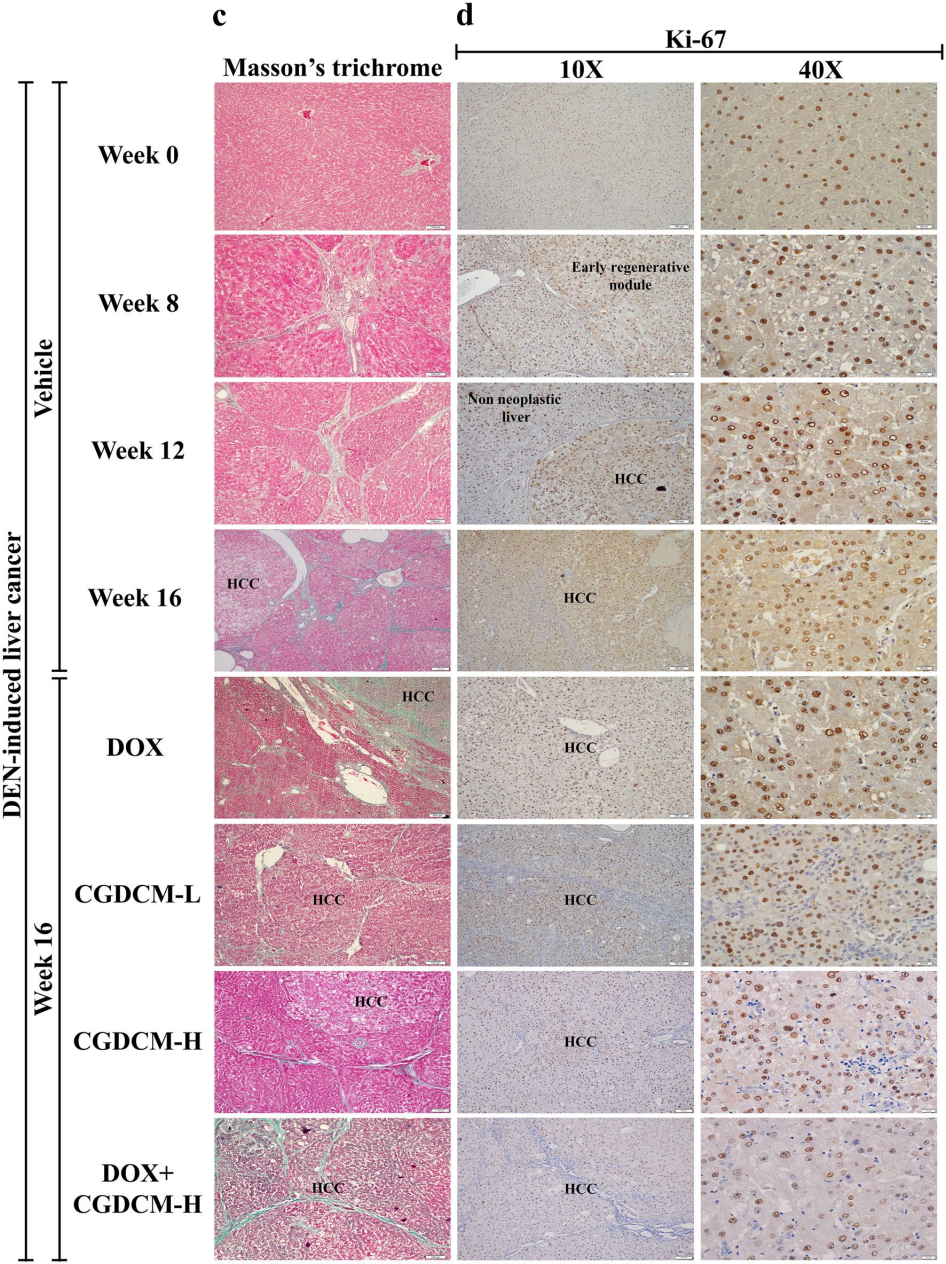
The effect of a combination of DOX and CGDCM extract from stem bark of *C. gigantea* on the suppression of tumors in DEN-induced liver cancer rats. DEN administration was performed for 8 weeks, and the treatments were performed for 4 weeks from week 12, which included DOX, CGDCM-L, CGDCM-H, and the combination of DOX with CGDCM-H. The vehicle group was treated with 0.8% DMSO. (**a**) Representative photographs of the macroscopic appearance of the liver. (**b**) Representative images of microscopic examination of liver tissues stained with hematoxylin and eosin (H&E), (**c**) Masson’s trichrome staining, and (**d**) immunohistochemical analysis of Ki-67 expression. Bars = 100 µm and 20 µm under × 10 and × 40 magnification visualized by light microscopy. CGDCM*, C. gigantea* dichloromethane extract; CGDCM-L, CGDCM low dose; CGDCM-H, CGDCM high dose; DOX, doxorubicin.

Before DEN administration at week 0, the liver was grossly normal and had a smooth capsular surface and homogeneous tan color. In representative photographs, numerous visible, pale, neoplastic-like nodules readily appeared on the liver surface of rats after the last DEN administration at week 8, progressed at week 12, and were obviously pronounced at week 16 in the vehicle group compared to week 0 (the first DEN injection), whereas treatment with DOX or CGDCM alone (from week 12 to week 16) showed less severity (Fig. 8a).

#### Microscopic examination

The multinodular architecture of the liver was histologically observed in H&E sections at week 8 (Fig. 8b). There was a loss in normal hepatic architecture with varying degrees of hepatocyte injury, including hepatocyte swelling, hydropic and vacuolar degeneration, and inflammatory cell infiltration. Classic HCC was diagnosed in most liver nodules at weeks 12 and 16 (the HCC area was focused at 40 × magnification). Neoplastic liver cells were predominantly arranged in a thick trabecular pattern and showed a high nuclear-cytoplasmic (N/C) ratio and pleomorphic nuclei. Tumor necrosis and hemorrhage were frequently observed, particularly at week 12 and in the vehicle group at week 16. Treatment with CGDCM-H alone reduced liver nodule size and number, as well as liver fibrosis, more than the other single treatment groups (DOX and CGDCM-L) and the combined DOX and CGDCM-H treatment group, when compared to the DEN-vehicle group at week 16.

#### Masson’s trichrome staining

Masson’s trichrome staining highlighted connective accumulation in the liver tissue (green color). The presence of connective tissue in nonneoplastic liver tissue was higher than the normal level from weeks 8 to 16 compared to the liver tissue at week 0 (Fig. 8c). Initially present at week 8, there was prominent or severely increased fibrosis in the portal tracts, with fibrous bridging and nodular formation, which was diagnosed as cirrhosis. Reduction in connective tissue accumulation in nonneoplastic liver tissue was observed in the group with CGDCM-H treatment alone.

#### Immunohistochemical analysis

Ki-67 antigen is a nuclear protein that is expressed by proliferating cells during the active cell cycle (G1, S, G2 and M phase)^3,45^. Increased Ki-67 protein expression in malignant tumors indicates significant proliferative activity and tumor aggressiveness. A disruption in cellular metabolism was connected to the inhibition of HCC development, resulting in reduced Ki-67 expression in the liver^33^. In comparison to that at week 0, increased nuclear expression of Ki-67 was seen from week 8 to week 16 after DEN injection. Nuclear Ki-67 expression was more prominent in cancer tissues than in surrounding noncancerous tissues (Fig. 8d, × 40 magnification presented). Reduced nuclear staining for Ki-67 was observed in the CGDCM-H-treated group compared to the DOX-, CGDCM-L-, and combined DOX and CGDCM-H-treated groups. These results indicated that CGDCM-H had a substantial inhibitory effect on cellular metabolism, resulting in a reduction in DEN-induced hepatic fibrosis and carcinogenesis in rats.

**Figure 9:**
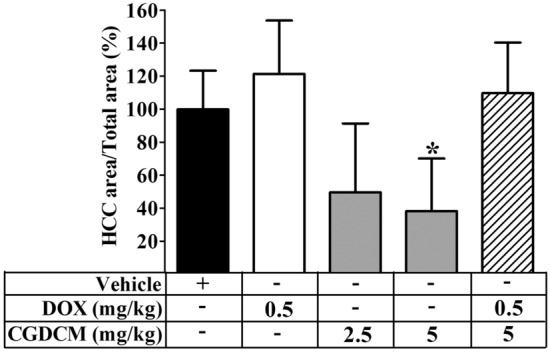
Quantitative examination of the prevalence of HCC. Liver H&E sections at the end of the treatments (week 16) were scanned by Zeiss Axio Scan Z1 (Zeiss, Germany) and measured the area by the METAVIR scoring system (ZEN blue 2.3). Histograms show the relative HCC area (%). The vehicle group was treated with 0.8% DMSO. DEN administration was performed for 8 weeks, and the treatments were performed for 4 weeks from week 12, which included DOX, CGDCM-L, CGDCM-H, and the combination of DOX with CGDCM-H. The data were analyzed using one-way ANOVA with Tukey’s HSD test and reported as the mean ± SD (n = 7), *p < 0.05 compared to the vehicle at week 0. CGDCM*, C. gigantea* dichloromethane extract; CGDCM-L, CGDCM low dose; CGDCM-H, CGDCM high dose; DOX, doxorubicin.

The findings of a quantitative examination of H&E staining to evaluate the prevalence of HCC in the study groups are depicted in Fig. 9. Following DEN administration, the area of HCC was significantly reduced in the CGDCM-H treatment group when compared to the DEN-vehicle group at week 16. Even though the CGDCM-L group had a reduced HCC area, these changes were not statistically significant when compared to the DEN-vehicle group at week 16. This result suggests that CGDCM-H therapy has anticarcinogenic properties.

### In DEN-induced HCC rats, a combination of DOX and CGDCM reduced hepatic inflammation

**Figure 10:**
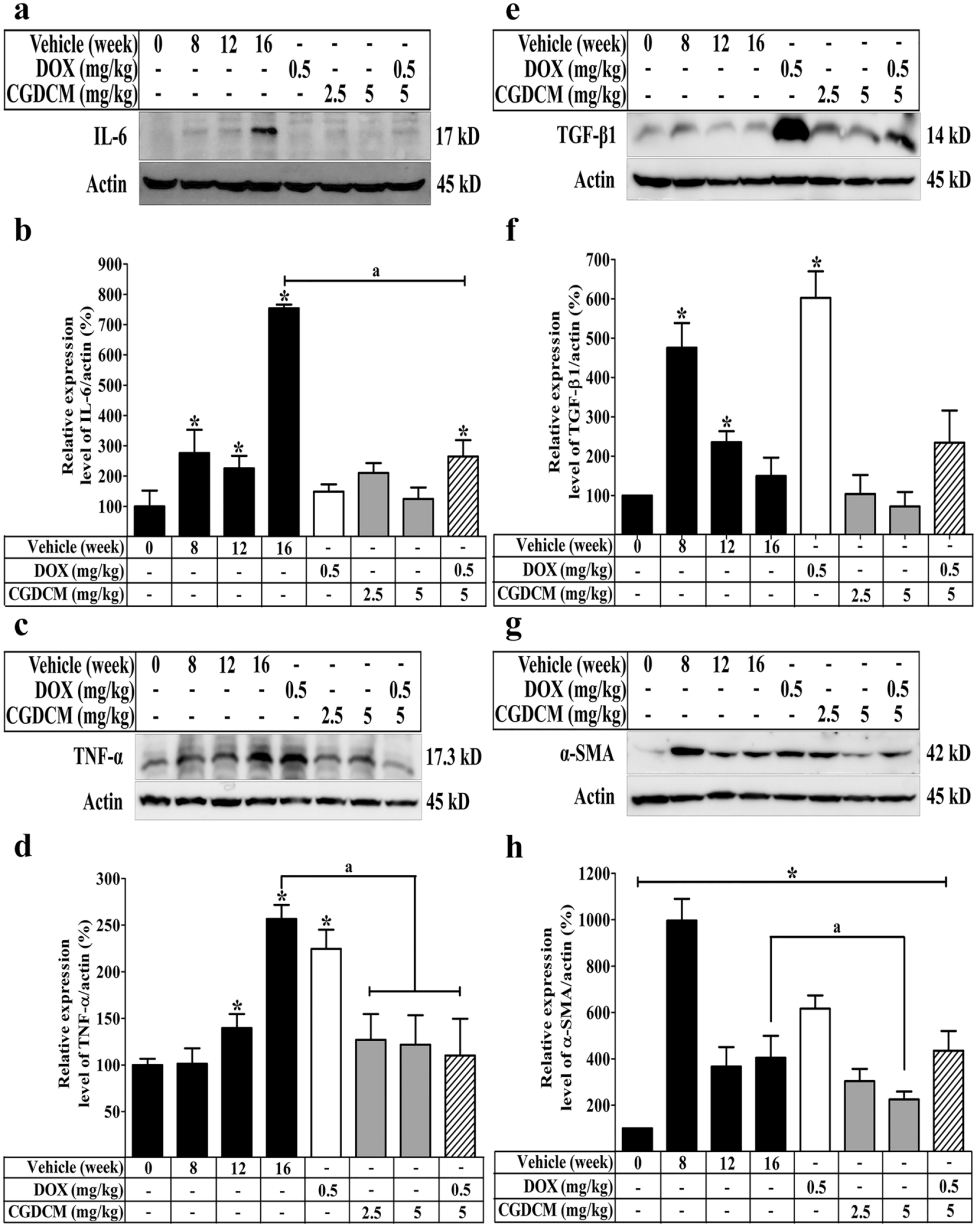
The effect of a combination of DOX and CGDCM extract from the stem bark of *C. gigantea* on the expression of inflammatory and fibrotic proteins in DEN-induced liver cancer rats. DEN administration was performed for 8 weeks, and the treatments were performed for 4 weeks from week 12, which included DOX, CGDCM-L, CGDCM-H, and the combination of DOX with CGDCM-H. The vehicle group was treated with 0.8% DMSO. Representative western blot images and quantitative analysis of the expression in a percentage of β-actin/protein expression from liver tissue of (**a**,**b**) IL-6, (**c**,**d**) TNF-α, (**e**,**f**) TGF-β1, and (**g**,**h**) α-SMA. The data are reported as the mean ± SD (n = 4) and were analyzed using one-way ANOVA with Tukey’s HSD test, *p < 0.05 compared to week 0. CGDCM*, C. gigantea* dichloromethane extract; CGDCM-L, CGDCM low dose; CGDCM-H, CGDCM high dose; DOX, doxorubicin.

The inflammatory process is triggered by DEN-induced liver damage, which leads to the development of liver cancer^46,47^. Oxidative damage caused by DEN exposure resulted in the activation of inflammation in the liver^48^. Inflammatory cytokines, including interleukin 6 (IL-6) and tumor necrosis factor alpha (TNF-α), are elevated in DEN-induced HCC rats^49^. The current study found that DEN considerably elevated IL-6 and TNF-α expression from week 8 to week 12 and greatly increased their expression in week 16 when compared to that in rats that were not given DEN (Fig. 10a–d). This result supported the suggestion that cellular damage causes inflammation in DEN-treated rats. Administration of DOX, CGDCM, or their combination reduced the expression of IL-6, although DOX had no influence on the level of TNF-α expression. Thus, following the inhibition of DEN-induced hepatic damage, CGDCM alone and combined with DOX reduced the inflammatory process, implying anticarcinogenic potential.

Furthermore, transforming growth factor beta 1 (TGF-β1) has been discovered as a critical marker for hepatic stellate cells (HSCs) transdifferentiating myofibroblasts to synthesize collagen to compensate for DEN-induced cell injury. Fibrosis is a major feature of liver cirrhosis, which contributes to the development of liver cancer^50–52^. TGF-β1 expression peaked at week 8 and then began to decline until week 16, when it was nearly equal to that of rats that were not given DEN. DOX significantly increased TGF-β1 expression. TGF-β1 expression was lower after 4 weeks of CGDCM treatment and the difference was not statistically significant when compared to the vehicle group at week 16. TGF-β1 expression was increased by combining DOX and CGDCM, but it remained lower than the peak level at week 8 (Fig. 10e,f).

Activated HSCs that have transformed into myofibroblast-like cells as a result of inflammatory responses to liver injury caused by DEN are represented by alpha-smooth muscle actin (α-SMA) expression in the cirrhosis stage^33^. We found that α-SMA expression was greater at week 8 and steadily decreased until week 16 when compared to normal rats at week 0. α-SMA expression was still higher in the DOX treatment group for 4 weeks than in the vehicle control group at week 0, whereas CGDCM alone or combined with DOX reduced α-SMA expression (Fig. 10g,h). Overall, the findings suggest that CGDCM alone or combined with DOX has anticancer activity by slowing the progression of hepatic fibrosis, which leads to a reduction in hepatocarcinogenesis.

### In DEN-induced HCC rats, a combination of DOX and CGDCM decreased liver fatty acid and ATP levels

**Figure 11:**
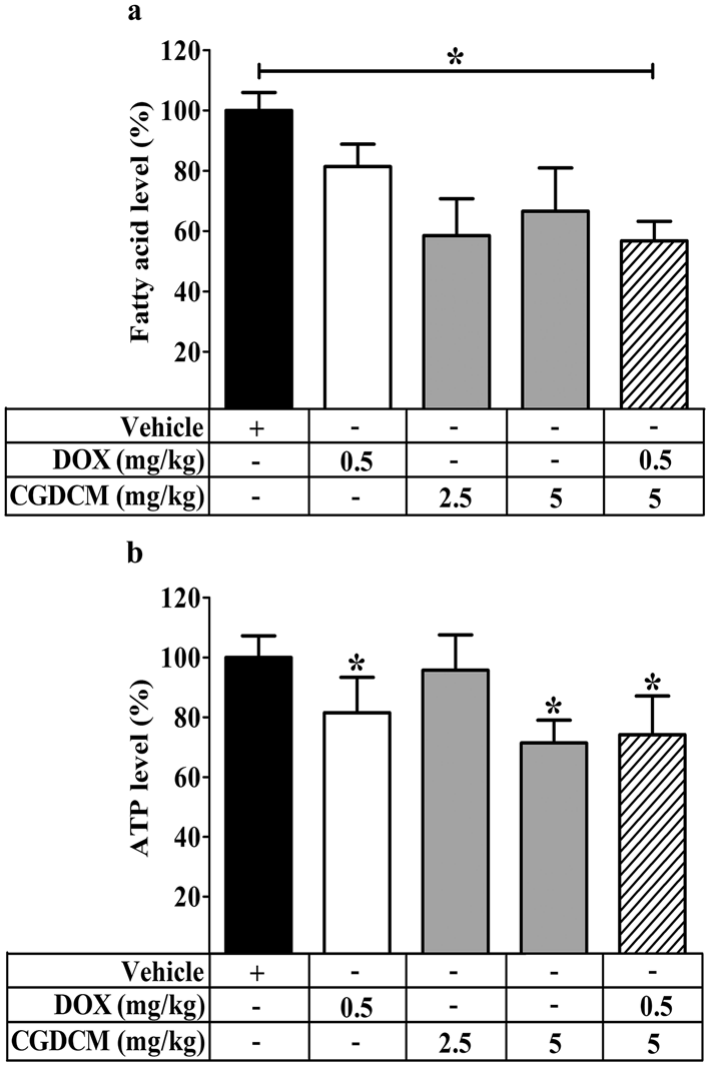
Combined effect of CGDCM extract from stem bark of *C. gigantea* on the production of fatty acid and ATP levels in DEN-induced liver cancer rats. DEN injection was performed for 8 weeks, and the treatments were performed for 4 weeks from week 12, which included DOX, CGDCM-L, CGDCM-H, and the combination of DOX with CGDCM-H. The vehicle group was treated with 0.8% DMSO. The percentages of (**a**) fatty acids and (**b**) ATP were measured in the liver tissue of rats at week 16. The data are reported as the mean ± SD (n = 3) and were analyzed using one-way ANOVA with Tukey’s HSD test, *p < 0.05 compared to week 0. CGDCM*, C. gigantea* dichloromethane extract; CGDCM-L, CGDCM low dose; CGDCM-H, CGDCM high dose; DOX, doxorubicin.

According to several lines of evidence, increased lipid biosynthesis and protein expression in the de novo fatty acid synthesis pathway promoted liver proliferation in chronic damage after DEN treatment^53^. Upregulation of fatty acid synthesis in response to DEN exposure supplied additional ATP for promoting hepatocarcinogenesis^54^. Thus, in the present study, we evaluated fatty acid and ATP generation in HCC induced by DEN. When compared to the DEN-induced HCC vehicle group, we discovered that DOX, CGDCM, and a combination of the two treatments significantly reduced fatty acid and ATP levels (Fig. 11a,b), indicating that the underlying mechanism of the anticancer effect is through the reduction in fatty acid and ATP synthesis.

### In DEN-induced HCC rats, a combination of DOX and CGDCM reduced liver apoptosis

**Figure 12:**
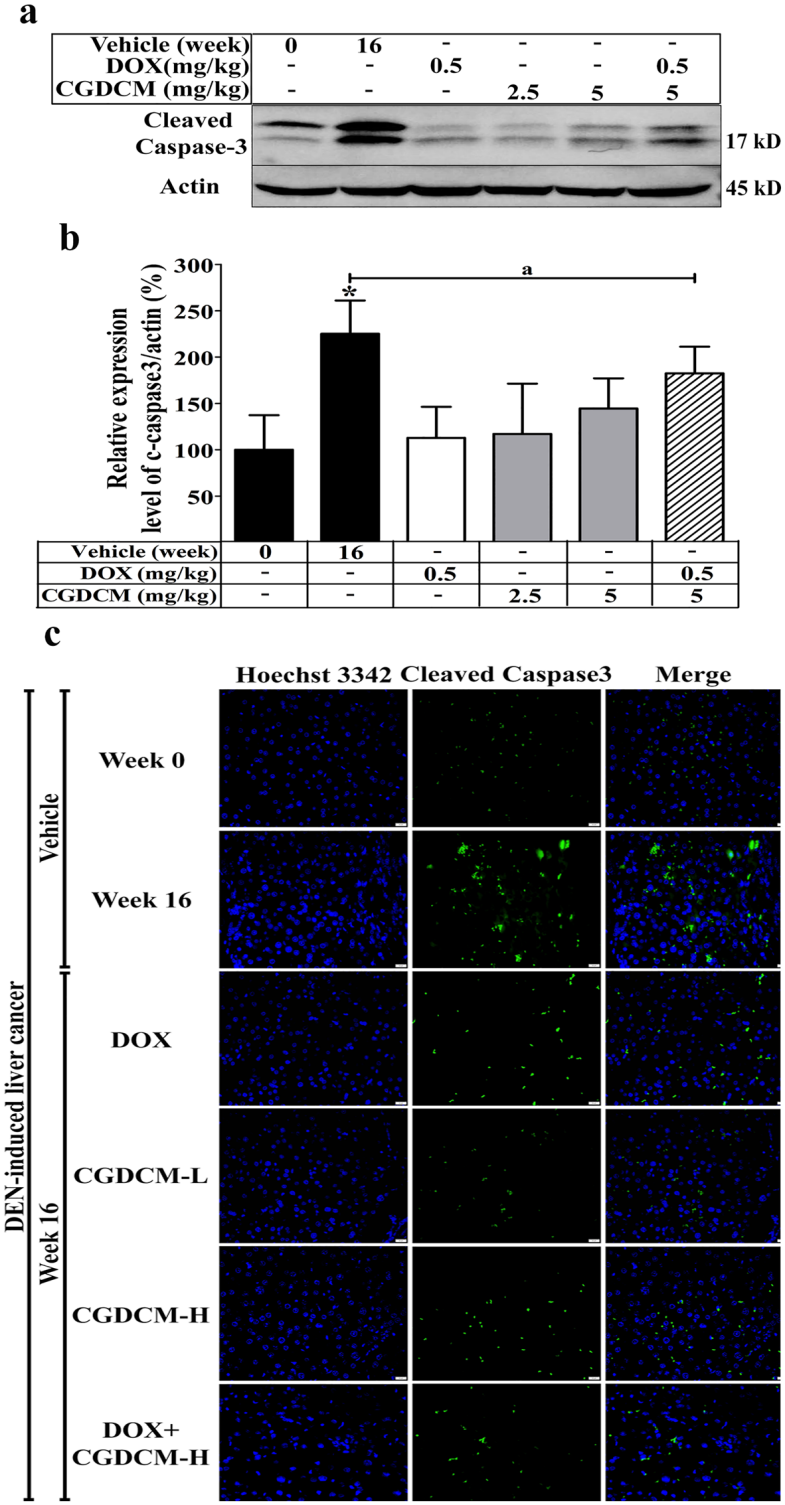
The effect of a combination of DOX and CGDCM extracts from stem bark of *C. gigantea* on the induction of apoptosis in DEN-induced liver cancer rats. DEN administration was performed for 8 weeks, and the treatments were performed for 4 weeks from week 12, which included DOX, CGDCM-L, CGDCM-H, and the combination of DOX with CGDCM-H. The vehicle group was treated with 0.8% DMSO. Representative images from the liver tissue by western blot analysis of (**a**) cleaved caspase-3 and (**b**) quantification of the expression as a percentage of β-actin/protein expression. (**c**) Representative images of cleaved caspase-3 expression visualized by fluorescence microscopy and counterstained with Hoechst 33342, bars = 20 µm. The data are reported as the mean ± SD (n = 3) and were analyzed using one-way ANOVA with Tukey’s HSD test, *p < 0.05 compared to week 0. CGDCM*, C. gigantea* dichloromethane extract; CGDCM-L, CGDCM low dose; CGDCM-H, CGDCM high dose; DOX*, * doxorubicin.

The mechanisms of apoptosis that underlie the anticancer effects of various anticancer treatment regimens against DEN-induced cancer progression in animals remain controversial. Increased apoptosis was identified in DEN-induced hepatotoxicity in rats^32,55^. Activation of the apoptotic process inhibits aggressive cell proliferation and the progression of cancer cells^3^. Sancho et al., found that inhibiting apoptosis slowed the progression of fibrosis and delayed the onset of liver cancer^56^. In DEN-treated rats, polyphenol, quercetin, and mulberry extracts exhibited anti-proliferative activity by decreasing apoptotic activity and protecting against hepatocarcinogenesis^55,57^. To evaluate the contribution of apoptosis to the anticancer efficacy of therapies for HCC, we examined the expression of cleaved caspase-3 in whole liver homogenates. Our findings revealed that in the DEN vehicle group, cleaved caspase-3 was upregulated, implying that apoptosis enhanced HCC formation. Rats treated with DOX, CGDCM, or a combination of the two drugs had significantly lower cleaved caspase-3 expression in the liver, as shown by western blotting (Fig. 12a,b) and immunofluorescence assays (Fig. 12c). This result suggests that inhibiting apoptosis protects against repeated liver damage and the development of HCC. However, our in vitro data, which revealed that DOX, CGDCM, and a combination of the two drugs inhibited HepG2 cell growth and invasion by enhancing apoptosis, contradicted the observed anticancer impact in DEN-induced HCC rats.

### Combinations of DOX and CGDCM had no toxicity on internal organs in DEN-induced rats

**Figure 13:**
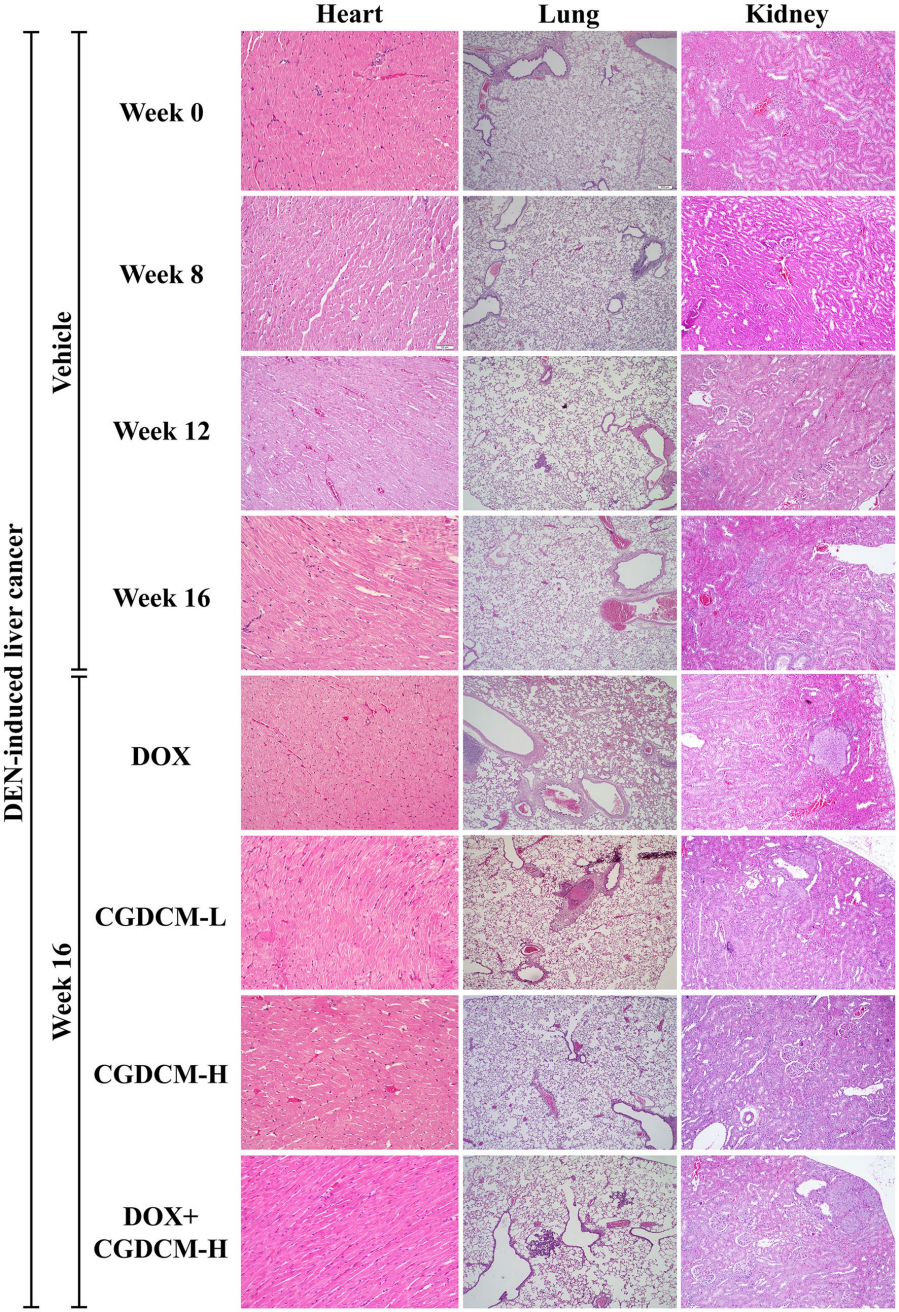

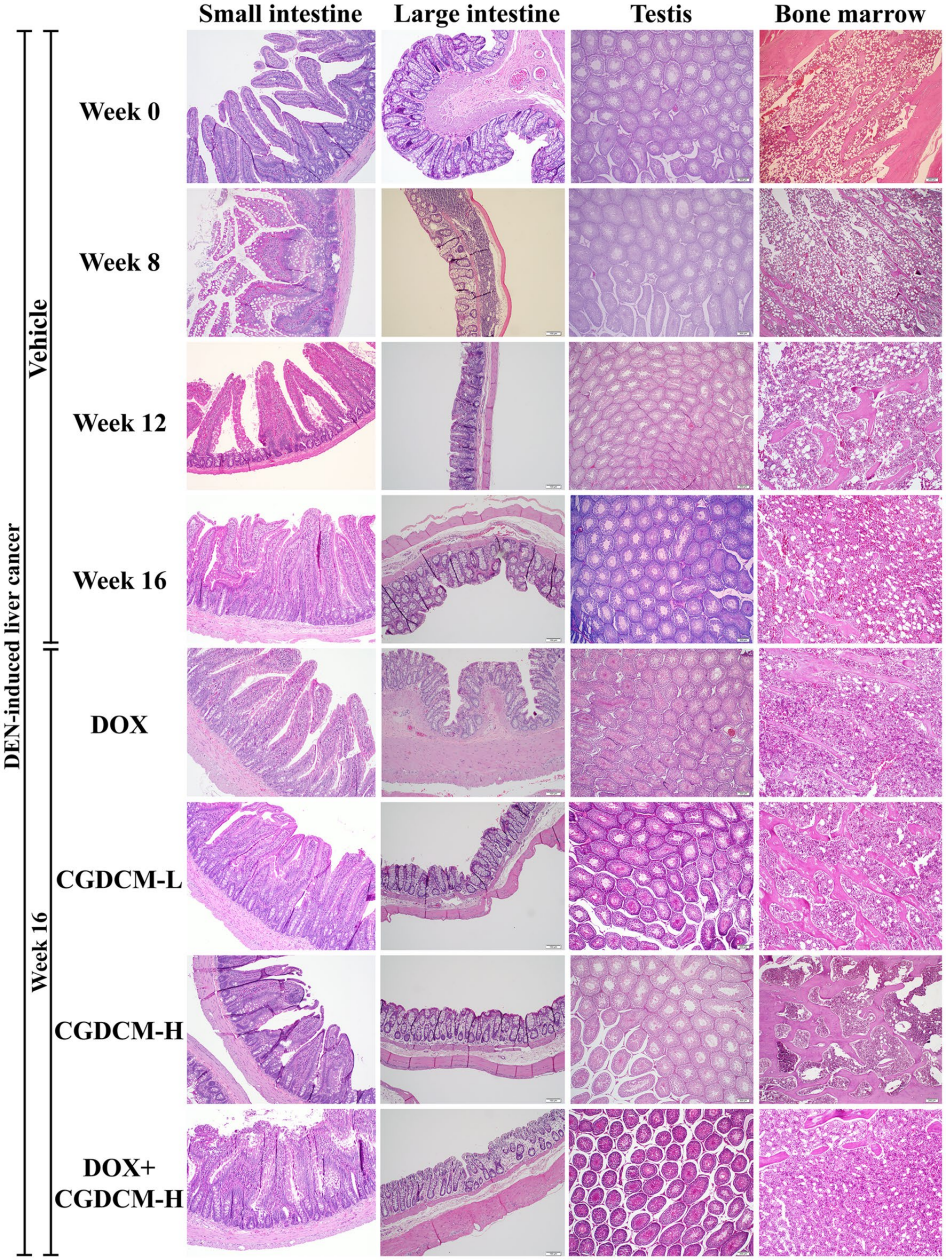
The toxicity in rat tissues in DEN-induced liver cancer rats. Representative histopathological sections of rat tissues were stained with H&E in DEN-induced liver cancer rats for 8 weeks, and the treatments were performed for 4 weeks from week 12, which included DOX, CGDCM-L, CGDCM-H, and the combination of DOX with CGDCM-H extracts from stem bark of *C. gigantea*. The vehicle group was treated with 0.8% DMSO. Scale bars = 200 μm, 100 µm, and 50 µm under × 4, × 10, and × 20 magnification. CGDCM*, C. gigantea* dichloromethane extract; CGDCM-L, CGDCM low dose; CGDCM-H, CGDCM high dose; DOX, doxorubicin.

The present study assessed the toxicity of DOX, CGDCM, and a combination of the two drugs in internal organs, including the heart, lung, kidney, small and large intestines, testes, and femur bone marrow, in DEN-induced HCC rats (Fig. 13). Additionally, we found foci of carcinogenesis in the kidney and lung of DEN-treated rats, implying that DEN toxicity is nonspecific. Thus, the treatment regimens had no deleterious effects on the rats' internal organs, indicating that *C. gigantea* stem bark extract administration in combination with DOX at a low dose is safe.

## Discussion

Liver cancer has become one of the main causes of cancer-related deaths worldwide due to a lack of effective treatments and a variety of causative factors, such as genetics and epigenetics^58–60^. Despite the fact that chemotherapeutic medications are effective therapies for HCC, their high toxicity, considerable side effects, and long-term drug resistance restrict clinical outcomes, resulting in an increasing death rate^61^. Several lines of evidence have been gathered to support potential HCC therapeutic regimens employing traditional medicinal plant extracts^6–14^.

*Calotropis gigantea* extracts have been shown to have anticancer activity in a range of cancer cells. *C. gigantea* whole plant extract exhibited cytotoxicity in A549 and NCI-H1299 non-small-cell lung cancer cells^62^. Ethanolic extracts from roots, flowers, and leaves had cytotoxic effects on T47D breast cancer cells^63^. *C. gigantea* leaf extracts were shown to suppress fibrosarcoma growth in mice induced by 7,12-dimethylbenz-(α)-anthracene by inducing apoptosis^64^. In Ehrlich ascites carcinoma tumor-bearing mice, methanolic and chloroform extractions of root bark^41^ and ethyl acetate extracts of *C. gigantea* flowers were observed to decrease tumor cell development^65^.

Several studies have suggested that cardiac glycosides, flavonoids, triterpenoids, and phenolic compounds isolated from *C. gigantea* contribute to the cancer therapeutic effects of the extracts. Cardenolides isolated from leaves and stems, including calactin, uscharin, afroside, calotoxin, gomphoside, and two unknown cardenolides, were found to decrease breast cancer MCF-7-cell proliferation^31^. Flavonoid and terpenoid chemicals from *C. gigantea* leaves were discovered to be cytotoxic against human colon cancer WiDr cells^18^. Calotropin isolated from leaves has been shown to induce apoptosis in SW480 cells^19^ and to inhibit cell proliferation in HCT 116 and HT-29 colon cancer cells^20^. Coroglaucigenin isolated from the roots of *C. gigantea* triggered senescence and autophagy in colorectal cancer cells^22^. Cardenolides isolated from *C. gigantea* stems and leaves were found to be highly cytotoxic to HeLa and human lung cancer cells^21^. Cardinolides (uscharin, 15*β*-hydroxyuscharin, 19deoxy-15*β*-hydroxyuscharin, 2ʺ-*oxo*-voruscharin, calactin, calotropin, gomphoside, and asclepin) isolated from latex and fruit evoked cytotoxicity in MCF-7 human breast cancer cells but not in MCF-10A human normal mammary epithelial cells^30^.

The results revealed that the percent yield of CGEtOH (3.3%) obtained from the maceration method at room temperature (27 ± 5 °C) was less than that in our previous report (5.6%)^66^ using ultrasonic-assisted extraction. CGEtOH was fractionated to divide the phytochemicals according to their solubilities in each organic solvent. CGDCM was expected to contain the most nonpolar components, which could be dissolved in dichloromethane. The CGDCM yield was quite high at 17.5%. According to previous reports, CGEtOAc may include moderately polar compounds and is projected to contain the maximum amount of cardenolides, a major group of active compounds in *C. gigantea*^30,67^. However, this fraction had the lowest yield of only 1.2%. CGW was the major fraction, with 68.4% yield.

Our present study indicated that all four tested samples, including CGEtOH, CGDCM, CGEtOAc and CGW, contained cardiac glycosides, triterpenoids, phenolic compounds, flavonoids, alkaloids and calactin with different magnitudes similar to those in a previous report by our group^66^. The total contents of phenolic compounds and flavonoids reported in this study corresponded to those found in *C. gigantea* flowers^16^. Astonishingly, CGW, which was expected to be the most polar fraction, showed the lowest phenolic content. This may be due to the distribution of phenolic compounds to other fractions by subsequent liquid–liquid partition. However, these results showed the same trend as a previous report^66^. Among the tested samples, CGDCM comprised the highest amounts of triterpenoids, flavonoids and calactin, while CGEtOAc contained the highest contents of cardiac glycosides and phenolic compounds. These two fractions were expected to contain nonpolar and moderately polar compounds, especially cardenolides such as uscharin, calactin, calotropin and calotoxin, which were the major cardenolides isolated from the ethyl acetate layer of the latex^30,67^ and the root bark of *C. gigantea*^68^. The IC_50_ values against HepG2 from the MTT assay in this study suggested that CGDCM was the most potent fraction, followed by CGEtOAc, which may be related to their variety and high amounts of chemical compositions. Altogether, CGDCM from *C. gigantea* stem bark extract is thought to have anticancer properties due to cardiac glycosides, triterpenoids, and phenolic phytochemical components. As a result, CGDCM was selected for further investigation into apoptotic activity against HepG2 cells, either alone or in combination with DOX.

The present study demonstrated that a combination of low concentrations of DOX at 0.5 µM (0.27 μg/mL) and CGDCM at 25, 50, and 100 µg/mL caused a stronger apoptotic impact than either treatment alone in HepG2 cells. DOX concentrations ranging from 1 to 100 µM have been reported to have potent anticancer activity against a variety of cancer cells, including cervical cancer HeLa cells^69^, murine breast cancer cells^70^, mouse E0771 breast cancer cells^71^, A-172 brain, glioblastoma and mammary gland, adenocarcinoma MCF-7 cells^72^, canine transitional cell carcinoma cells, canine osteosarcoma cells, canine hemangiosarcoma cells^73^, and human liver cancer BEL-7404 cells^74^. However, drug resistance and the occurrence of systemic adverse effects on the heart, liver, kidneys, and testis associated with DOX treatment, as well as its poor application due to its short half-life, limit its utility^14,24–26^. To overcome these barriers and achieve therapeutic success, some studies recommended a combination regimen of medicinal plant extracts and DOX. Danthron, a compound derived from the root and rhizome of *Rheum palmatum* L., sensitized human pancreatic cancer cells to DOX-induced cell death^75^. A synergistic anticancer effect was achieved by combining DOX with the cinnamaldehyde compound found in cinnamon bark (*Cinnamomum spp*., Lauraceae)^76^. Therefore, combination therapy that uses a lower dose of medications and extracts than monotherapy may therefore be an effective therapeutic method for enhancing anticancer activity, overcoming drug resistance, and minimizing adverse side effects.

The present study found that a combination of low doses of DOX and CGDCM generated cytotoxicity in HepG2 cells by inducing apoptosis accompanied by suppressing ATP production. Lower levels of ATP have been identified as a critical tumor-suppressing mediator for antitumor activity^34^. Many extract components from natural plants have anticancer potential correlated with ATP production, which is a significant modulator of apoptotic triggering. Apoptosis was discovered in cancer cells after treatment with polyphenol curcumin derived from *Curcuma Longa* L. (Zingiberaceae), which depleted ATP production and the oxygen consumption rate^35^. Moreover, the flavonoid wogonoside isolated from the root of *Scutellaria baicalensis Georgi* induced apoptosis accompanied by mitochondrial dysfunction, which was mediated by decreased ATP levels activating AMPK/mTOR signaling in human non-small-cell lung cancer A549 cells^36^. In addition, flavonol aglycone isorhamnetin constituents in many traditional medicinal herbs evoke lethal action in human bladder cancer cells by decreasing ATP levels^37^. It was reported that the release of cytochrome C and the triggering of apoptosis in cancer cells are caused by a decrease in cellular ATP due to the repression of glycolysis and oxidative phosphorylation. This metabolic inhibition was caused by the separation of hexokinase from voltage-dependent anion channel 1 on the outer mitochondrial membrane^33^. However, the mechanism by which ATP causes apoptosis in cancer cells, as demonstrated by *C. gigantea* extracts, is still unidentified. The mechanisms of ATP production inhibition correlated with apoptosis induction were reported for ginsenoside obtained from ginseng. Decreasing ATP production by ginsenoside was associated with inhibiting glucose uptake and the glycolysis pathway, resulting in a cell growth inhibitory effect in hepatocellular carcinoma cells. According to that finding, ginsenoside enhanced the ubiquitination degradation of hypoxia-inducible factor-1α, a critical transcription factor that regulates metabolism pathways in cancer cells^38^. According to several studies, anticancer drugs dissipate the mitochondrial membrane potential, which is recognized as the primary contributor to apoptotic cell death in cancer cells. This means that OXPHOS is incapable of producing ATP^77–79^. Certain cancer cells were discovered to switch to glycolysis, but they were unable to counteract an ATP deficit and the effect of oxidative stress, which increased the energy deficiency by inhibiting fatty acid oxidation in mitochondria^77^.

Reactive oxygen species (ROS) are important regulators of apoptosis in many cancer cells. Chemical extracts from several plants promoted anticancer activity by inducing the generation of ROS^40,80–83^. In the non-small-cell lung cancer cell lines A549 and NCI-H1299, ROS generation after treatment with ethanolic extracts from the whole plant of *C. gigantea* was shown to mediate apoptotic cell death^62^. Coroglaucigenin isolated from the stems and leaves of *C. gigantea,* inhibited antioxidant molecules, resulting in enhanced ROS generation^21^. The production of ROS has been identified as a crucial intracellular ATP regulator for apoptosis in colon cancer^84^ and ovarian cancer cells^85^.

We generated a model of chemically induced liver cancer using DEN, which is a well-established carcinogenic effect in rodents. DEN is primarily metabolized in the liver by cytochrome P450, particularly CYP2E1 enzymes, creating hazardous alkylating metabolites that have the potential ability to alkylate and damage the DNA of hepatocytes^86^. Cellular damage is followed by inflammation, where cell death is repeatedly enhanced, causing compensatory regeneration of neighboring living cells and eventually resulting in liver cancer or HCC formation in almost all animals similar to that found in humans^43,57,58,87,88^. It was suggested that DEN-induced inflammatory responses contribute to the progression of compensatory proliferation of hepatocytes to become carcinoma^87^. Damage-associated molecules, including ATP released from damaging hepatocytes, trigger the recruitment of inflammatory cells to a repeat cycle of hepatocyte damage, facilitating the proliferation of hepatocytes and carcinogenesis formation^89^.

Hepatic damage caused by DEN was probably influenced by the oxidative damage process of DEN metabolism^47,90^. Increased production of ROS due to DEN metabolism contributes to an imbalance in cellular oxidative and antioxidative defense capacities^91^. Increased free radicals were detected at least 1 h after DEN exposure. Not only upregulation of ROS production but also suppression of antioxidant molecules, including superoxide dismutase (SOD), catalase (CAT), and peroxidase, by DEN enhanced the repeated cycle of oxidative DNA damage and induced necrotic cell death^48,88,92^. Enhanced consumption and exhaustion of antioxidant enzyme activities, including CAT, glutathione reductase (GR), glutathione S transferase (GST) and reduced glutathione (GSH), was found after DEN administration^93^. It has been shown that higher amounts of malondialdehyde (MDA) and reactive oxygen and nitrogen species and lower levels of GSH and antioxidant enzymes SOD and CAT are found in parenchymal cells (microsomes, mitochondria, and peroxisomes) in DEN-induced hepatic injury^43,47,94^. The overutilization of excessive free radicals and oxidative stress production generated by DEN in the liver resulted in a depletion of antioxidant defensive action^57,90,93^. However, some contradictory evidence suggests that DEN exposure enhanced cellular redox scavenger levels, including SOD, CAT, GST and GSH levels, indicating redox homeostasis compensation to encounter increased lipid peroxidation and DNA damage during malignancy development^44^. DNA adduct generation and oxidation of RNA and DNA induced by ROS after DEN treatment have been postulated to be significant mechanisms of HCC development^90^. Interfering with the DNA repair pathway can aggravate DNA damage, leading to a decrease in antioxidant gene expression in the liver, which is responsible for scavenging oxidative molecules^93^. Thus, enhanced ROS generation by DEN appears to be a key factor in liver injury leading to HCC development^95^.

The present study found that after 8 weeks of DEN treatment, the rats were allowed another 4 weeks to develop cancer before being treated with the extracts for another 4 weeks. The liver had a multinodular architecture, as well as an increased region of tumor foci, according to histological investigation. The progression of hepatic nodules of various sizes was obviously visible after 16 weeks of DEN injection, suggesting liver damage that contributed to the development of liver cancer, as reported previously^33,96^. We found that DEN had no effect on rat body weight, which is consistent with previous studies demonstrating that animals’ food and water intake did not decrease during 16 weeks of DEN treatment^43,94,96^. However, some evidence showed a lack of appetite due to a liver tumor after 20 weeks of DEN treatment^90,96^. After DEN treatment, an increased liver-to-body weight ratio suggested the development of liver multinodular formation^97,98^. These phenotypic changes were correlated with a rise in cell-destructive markers, such as AST and ALT, which leaked from hepatocytes, particularly following DEN therapy at weeks 3 to 8, suggesting established hepatic damage, as previously reported^94,99–101^. After the last DEN injection at week 8, the levels of AST and ALT declined, but they remained higher than those in the control group (week 0). Treatment with CGDCM exhibited a propensity to reduce elevated levels of AST and ALT, as well as visible restoration of liver histology, fewer tumors, and a lower liver-to-body weight ratio, showing that CGDCM may protect against DEN-induced liver toxicity. In DEN-induced cirrhosis and HCC, suppression of AST and ALT levels has been proposed as a key marker of anticancer activity^6,8,9^.

The release of profibrogenic factors by inflammatory and Kupffer cells, including transcription growth factor, cytokines, and chemokines, has been related to the presentation of excessive ROS by DEN, which accelerates liver fibrosis and ultimately HCC progression^49,51,102^. When the liver is repeatedly injured by DEN-induced oxidative stress, inflammatory cells and parenchymal cells release large amounts of cytokines and chemokines, which activate nuclear transcription factor kappa B (NF-κB), amplifying liver inflammation and increasing aggressiveness^94,103,104^. IL-6 and TNF-α can induce NF-κB phosphorylation for the production of inflammatory factors^98^. Hepatocyte growth factor (HGF) produced by stellate cells and the inflammatory cytokines IL-6, TNF-α, and TGF-β1 released from dead hepatocytes have been reported to activate Kupffer cells, causing liver damage and accelerating hepatocarcinogenesis^46,47,98,99,105^. Inflammatory responses are produced in nearby hepatocytes by IL-6 and TNF-α activation, worsening a vicious chemical communication loop between hepatocyte damage and Kupffer cells, culminating in enhanced hepatocyte proliferation^49,101,106^. Despite the fact that hepatocyte survival is driven by inflammation, hepatocyte proliferation is enhanced, leading to hepatocarcinogenesis. Inflammatory cytokines trigger mitogen-activated protein (MAP) kinases, including the cJun NH2-terminal kinase (JNK) signaling pathway, which is implicated in cell cycle progression and neoplastic transformation^97^.

In the current study, IL-6 and TNF-α were elevated after DEN injection starting from the onset of DEN exposure. Therapy with CGDCM resulted in a decrease in IL-6 and TNF-α expression, implying that the inflammatory signaling cascade is suppressed, slowing cancer progression. The existence of a positive feedback link between damage-associated molecules generated by damaged hepatocytes and inflammatory responses exacerbated irreversible liver damage, resulting in fibrotic/cirrhotic and hepatic carcinogenesis activation^88,97^.

TGF-β1 was found to be a key facilitator of malignant start in the early stages of HCC formation as a result of persistent and repeated liver injury by DEN, activating quiescent HSCs to synthesis matrix during the fibrosis stage of HCC development^103,107^. TGF-β1 from hepatocytes triggers HSCs to transform into myofibroblasts, which synthesize matrix proteins and prevent matrix degradation, resulting in the formation of the net fibrotic stage through the TGF-β/Smad pathway^50,108^. TGF-β1 is then expressed by activated HSCs, triggering the inflammatory-induced fibrosis cascade^102^. IL-6 and TGF-β1 also operate as positive stimulators of the cytoplasmic transcription factor STAT3 in HSCs, promoting fibrosis by stimulating the synthesis of fibrotic marker proteins. Cirrhosis and carcinogenesis are the results of long-term fibrosis^109^. Upregulation of TGF-β1 has been identified as a wound healing process that protects against the spread of hepatocyte proliferation associated with genetic abnormalities following injury^51,52^. However, during the activation of HSCs, the canonical Wnt pathway is upregulated, which targets several genes encoding proteins for promoting fibrogenesis and carcinogenesis^110^. HSCs activated by DEN treatment were observed to enhance α-SMA levels, a hallmark of this activation, in myofibroblasts, indicating the advancement of cirrhosis and the progression of cancer stromal cells^33,49^. Hepatic fibrogenesis is caused by increased α-SMA expression and extracellular matrix protein synthesis by HSCs, including collagens, proteoglycans, and glycoproteins^106,111^. In an in vitro model of activated HSCs, TGF-β1 upregulated the expression of α-SMA, collagen I, and III^112^.

In our investigation, repeated inflammation caused by DEN in liver cells resulted in an increase in IL-6 and TNF-α, which are responsible for repairing damaged hepatocytes. IL-6 and TNF-α enhanced the release of TGF-β1 to activate HSCs and cause fibrosis, suggesting that TNF-α and TGF-β1 upregulation occurred prior to liver fibrosis induction^113^. Our findings revealed a decrease in TGF-β1 and α-SMA expression after the first 8 weeks of DEN injection, while IL-6 and TNF-α expression remained elevated until week 16 after DEN administration was discontinued. The relevance of these changes, however, remains uncertain. We postulated that the specificity of DEN may be responsible for the alteration in TGF-β1 and α-SMA expression patterns. After CCl_4_ injection-induced liver fibrosis, TGF-β1 expression followed a similar pattern, rising for 3 days before declining on Day 5, which was associated with the pattern of ALT and AST levels, which peaked on Days 1 and 3 and then decreased on Day 5^114^. According to Ding et al., the inflammatory stage of DEN injection was found in the first 6 weeks, the fibrosis stage was found in the 10th week, and the HCC stage was found in the 20th week. TGF-β1 and α-SMA levels appeared to fall when the DEN injection was stopped at week 11, while IL-6 and TNF-α levels remained elevated at week 20^115^.

It is worth noting that the mechanism that controls TGF-β1 expression differs from that which controls IL-6 and TNF-α. Monocytes and macrophages produce IL-6 and TNF-α in acute and chronic liver injury, while monocyte-derived macrophages generate TGF-β1 during persistent inflammation-induced liver fibrosis^108,116,117^. During the first 2 weeks of fibrosis development by CCl_4_, TGF-β1 and its downstream signal molecule, phospho-Smad2, were elevated and then reduced with time (until week 7). TGF-β1-induced HSC activation resulted in an increase in α-SMA levels, which is a downstream fibrotic factor^56^. IL-6 and TNF-α drive the transition of liver cells from the G0 to the G1 phase of the cell cycle, which is important in the early phases of liver regeneration. TGF-β1 is not required for the termination of regeneration because the rate of regeneration slows when the liver returns to its normal capacity^118^.

The present results showed that DEN effectively promoted liver fibrosis with a correlation to collagen deposition, whereas CGDCM reduced fibrosis appearance with a link to suppression of TGF-β1 and α-SMA protein expression, implying that hepatic fibrosis was interrupted. TGF-β1 downregulation has been demonstrated to be an indicator of antifibrotic effectiveness in a previous study. When the natural bioflavonoid morin was used to treat DEN-induced fibrosis, HSCs were unable to produce matrix components^50^.

Our findings revealed that CGDCM treatment decreased the levels of the liver proliferative marker Ki-67 in DEN-induced HCC rats, implying anti-proliferative activity against liver cancer. DEN has been reported to enhance the proliferation and initiation of hepatocyte tumors by activating the G1/S phase of the cell cycle^96,119,120^. The enhanced proliferation of liver cells after DEN treatment supports compensatory responses for the development of primary HCC following DNA damage and necrotic cell death in the liver^3,88,121^. Increased expression of the signaling proteins Akt and ERK has been linked to the development of liver cancer in response to DEN exposure^97^. Reduced Ki-67 expression has been associated to a reduction in cell metabolism as an anticancer activity in DEN-induced animals with HCC^33^.

Although HCC cells require a lipid-rich environment or endogenous synthesis to survive, excess free fatty acids in the liver are a major cause of liver inflammation, which promotes the progression of hepatic cancer^122,123^. After DEN exposure, there was an increase in liver fatty acid accumulation, cholesterol, and C16 to C18 fatty acid elongation, which resembled nonalcoholic steatohepatitis-related hepatocarcinogenesis^124^. In DEN-injected mice, a high-fat diet accelerated the development of HCC^125^. Higher levels of saturated and poly-unsaturated fatty acids promoted carcinogenesis in DEN-treated animals^126–128^. Previous studies have reported that ROS activation and tumor TNF-α production are accompanied by an elevation of free fatty acids and cholesterol accumulation in mitochondria, which leads to liver damage and HCC development^124,129^. Upregulation of stearoyl-CoA desaturases results in the production of monounsaturated fatty acids (MUFAs), palmitoleate, and oleate, which have been linked to fibrosis synthesis in HSCs and liver tumor-initiating stem cell-like cells in the formation of alcoholic liver tumors^110^.

We explored the mechanism of the downregulation of lipogenesis, which was responsible for the anticancer activity of extracts from *C. gigantea* stem bark. The anticancer activity of CGDCM extracts was linked to a suppression in fatty acid levels in DEN-induced liver cancer. Carcinogenesis caused by DEN exposure has been demonstrated to be inhibited by a reduction in liver fat formation^33^. The prevention of liver cancer caused by DEN was shown to be mediated by a decrease in inflammatory cytokines and adipocyte shrinkage, as seen by an increase in serum glycerol and fat loss^122^. Hepatic fibrosis and carcinogenesis were reduced when fatty acid production was inhibited^110^. In line with previous research results, our findings revealed that CGDCM activity along with a reduction in fatty acid levels in the liver played a key role in the anticancer effects in DEN-induced HCC.

According to the findings of this investigation, significant apoptosis was found during DEN-induced HCC. The application of DEN caused repeated cellular damage and apoptosis, which aggravated inflammation through autocrine and paracrine mechanisms, resulting in increased compensatory hepatic overproliferation^43,96,97^. As a result, reducing DEN-induced liver injury and carcinogenesis by suppressing the inflammatory response has been discovered as a promising anticancer approach^9,10,12,13^. The conversion of cells to secondary necrosis was enhanced by increased apoptosis, which induced considerable inflammatory responses^3,56,88,91^. Increased apoptosis during the cell damage stage triggers inflammatory signaling, which is a key cause of hepatocarcinogenesis^130^. The release of proinflammatory cytokines such as IL-6, TNF-α, and TGF-β1 from hepatic macrophages causes apoptosis, which worsens liver inflammation-induced malignancy^56,97,131,132^. A previous study reported that upregulation of microRNA-34a by CCl_4_-induced liver fibrosis increased hepatocyte apoptosis, which participated in HSC activation with enhanced synthesis of α-SMA, TGF-β1, and collagen I, resulting in liver fibrosis^114,133^. As a result of failure in response to repeated liver injury by regeneration of hepatocytes to replace the damaged apoptotic cell, many hepatocytes eventually undergo apoptosis. HSCs and other nonparenchymal phagocytose apoptotic bodies of dead hepatocytes, resulting in the activation of HSCs and acceleration of the liver fibrosis process^134^. The uptake of apoptotic bodies by macrophages and HSCs activates TGF-β1 production, indicating a link between apoptosis and liver fibrosis activation in hepatocarcinogenesis^135^.

In our investigation, an increase in hepatic apoptosis after DEN administration was associated with a deterioration of the cell damage-induced inflammatory response, which has been linked to the development of hepatocellular carcinoma. Treatment with CGDCM decreased apoptosis, indicating that CGDCM inhibits the positive feedback of cell damage and carcinogenesis. It was reported that quercetin^57^ and mulberry water extracts^55^ showed a decrease in apoptosis activity and protective effect against hepatocacinogenesis in DEN-treated rats. In addition, the anticancer properties of salirasib and sorafenib therapy reduced apoptosis in tumoral and nontumoral tissues of rats given DEN^136^. *Punica granatum* (pomegranate) peel and seed oil extracts, which are rich in flavonoids and polyphenolic compounds, also inhibited caspase-3 activity in rats with DEN-induced liver damage^7^. Administering Myrrh from the stem of *C. molmol* (Nees) Engl. (Burseraceae) suppressed apoptosis, preventing DEN-induced hepatocarcinogenesis in rats^137^. According to Sancho et al., suppressing apoptosis slowed the progression of fibrosis and diminished the initiation of liver cancer^56^.

The discrepancy between the apoptotic response of CGDCM on HepG2 cells and DEN-induced rat HCC could be explained by several research hypotheses. The apoptotic activity of quercetin in HCC induced by DEN was observed at concentrations of 10–25 μM, while higher concentrations of 50–250 μM generated proapoptotic action, suggesting that the apoptotic response may be concentration dependent^57^. Following mulberry extract administration, apoptotic induction was increased in liver cells injured by DEN, while apoptosis was decreased in tumor foci of DEN-induced liver cancer^55^. According to Velasco-Loyden et al., treatment with IFC-305, an adenosine derivative drug, decreased nodules in DEN-treated rats independent of enhanced apoptosis. Changes in the DNA biosynthesis, HGF, Ras/MAPK and PI3K pathways that regulate liver carcinogenesis and cell cycle regulation may be primarily responsible for the reduction in cell proliferation^92^. In addition, apoptosis resistance may have contributed to the tumoral phenotypic change suggested by the anticancer effects of salirasib and sorafenib in DEN^136^. As reported for polyphenols, pomegranate peel and seed oil extracts^7^ and Myrrh from the stem of *C. molmol* (Nees) Engl. (Burseraceae)^137^, the antioxidative capabilities of plant extracts may have contributed to a reduction in apoptosis in DEN-induced HCC.

However, the abovementioned hypotheses could possibly be explained by the antioxidant and pro-oxidant properties of plant extracts. Several in vivo and in vitro studies indicate that *C. gigantea* has an antioxidant effect. The antioxidant reserve of the liver is essential for the detoxification of the vast majority of alkylating chemicals, including DEN. In accordance with earlier findings, antioxidant levels were found to decrease following administration of DEN. Posttreatment with *C. gigantea* extracts may increase the amount of antioxidants, a finding consistent with those of other plant extracts. Due to the antioxidant nature of the extracts, they neutralize the free radicals generated by DEN and prevent the cells from further oxidative stress. We concluded that the anticancer action of CGDCM involved an increase in apoptosis in HepG2 cells but a decrease in DEN-induced apoptosis in rat HCC cells. Additional mechanistic investigations are required to better comprehend the mechanism through which CGDCM inhibits apoptosis in DEN-induced liver cancer.

Despite the anticancer properties of DOX, its negative effect on a variety of cells limits its clinical application in the treatment of HCC. Treatment with 2 mg/kg DOX once a week for 4 weeks showed abnormalities in the heart, kidneys, liver, and testis, as well as degenerative functional alterations^24^. DOX therapy caused oxidative stress, which was linked to cardiotoxicity and hepatotoxicity in a dose- and time-dependent manner^14,25,26,138^. We discovered that DOX had no effect on reducing AST and ALT levels following DEN-induced HCC, which is consistent with prior findings that doxorubicin caused hepatotoxicity and elevated liver AST and ALT levels. The architecture of the hepatocytes did not improve after treatment with DOX^101^. We also found that DOX suppressed IL-6 expression, whereas TNF-α expression remained unaffected. Additionally, TGF-β1 and α-SMA protein expression remained elevated after DOX treatment. The presence of high levels of TGF‑β and α-SMA was correlated with liver fibrosis found in DOX treatment, implying that cirrhosis is an unfavorable impact of DOX during the suppression of HCC produced by DEN.

TNF-α levels in cardiomyocytes increased in DEN-treated mice after DOX administration at 5 mg/kg for 4 weeks, indicating severe cardiotoxicity and cardiac hypertrophy^139^. After a single injection of 20 mg/kg DOX was given to mice for 72 h, blood TNF-α was likewise elevated, causing muscle weakness^140^. DOX therapy resulted in increased TNF-α production, which was linked to mitochondrial respiration inhibition, implying neurotoxicity in brain tissue^141^. TNF-α and IL-6 levels were not restored to baseline in mice after receiving 2 mg/kg/day DOX administration for seven days prior to HepG2 cell-induced HCC for three consecutive days^101^. The liver-to-body weight ratio was the same in the DOX treatment group as it was in the DEN-induced HCC group in our investigation, which is consistent with the results of earlier research. However, hepatocyte necrosis and inflammatory cell infiltration were still observed^142^. TGF‑β1 has been reported to regulate myocardial fibrosis generated by DOX treatment by promoting cell growth, proliferation, and differentiation of cardiac fibroblasts^143^. In addition, TGF‑β and α-SMA were also shown to modulate kidney fibrosis produced by DOX therapy^144^.

It was hypothesized that the therapeutic and harmful effects of DOX were proportionate to increased blood concentration when DOX (7 mg/kg) was combined with ADI Z52020236, which comprises extracts of *Astragali Radix*, *A. senticosus*, *Ginseng Radix*, and *Mylabris*^145^. DOX and verapamil combined as an adjuvant treatment, on the other hand, improved anticancer activity in HepG2 cells, resulting in a positive outcome and reducing DOX's negative side effects^139^. Our findings demonstrated that combining CGDCM with a low dose of DOX (0.5 mg/kg) improved the anticancer activity of DOX by lowering inflammation-induced liver fibrosis and hepatocarcinogenesis, which was comparable to what was observed in the treatment of DEN-induced HCC by CGDCM. More research is needed to fully comprehend the synergistic effect of DOX and CGDCM, as well as the negative consequences of DOX. We discovered that DEN caused harm to the kidneys and lungs, implying that DEN is nonspecific. Not only the live but also the kidney and lungs were vulnerable to the cytotoxicity of DEN^91,96^.

In conclusion, our results demonstrate that the extract from the stem bark of *C. gigantea* exhibited potent anticarcinogenic effects against DEN-induced hepatic cancer, including a reduction in apoptosis-induced cancer progression. In DEN-induced hepatocarcinogenesis, our findings also showed that combining CGDCM with a low dose of DOX (0.5 mg/kg) enhanced the anticancer activity of DOX. To completely comprehend the implications of CGDCM and its combination with DOX for use in cancer therapy regimens, further investigation is needed.

## Materials and methods

### Preparation of *C. gigantea* stem bark extracts

#### Plant material

*Calotropis gigantea* (L.) Dryand stem bark (200 kg) was collected from January 2017 to January 2020 from the Thoen District, Lampang Province, Thailand (latitude/longitude: 17°36/′9″ N/99°12′50″ E). The bark was shade-dried at ambient temperature (35 ± 7 °C) to obtain 42 kg of dried stem bark (21% yield of fresh bark). The dried stem bark was powdered using a blender and stored in an airtight plastic bag at room temperature (30 ± 5 °C) for further extraction. A voucher specimen (No. 005191) of the plant used in this study was authenticated by Dr. Pranee Nangngam, a taxonomist, and deposited for reference at the PNU Herbarium, Department of Biology, Faculty of Science, Naresuan University, Phitsanulok, Thailand. Plant collection and the use of the collected plants for research purposes were approved by the Department of Agriculture, Ministry of Agricultural and Cooperatives, Thailand, according to the Plant Varieties Protect Act B.E. 2542 (1999) Section 53 under permission number 0278. All plant methods were performed in accordance with relevant guidelines and regulations.

#### Extraction

The *C. gigantea* stem bark powder (19.8 kg) was extracted with 95% ethanol (the ratio of the plant powder to 95% ethanol was 1 g:10 mL) by maceration at room temperature (27 ± 5 °C) for 48 h each time. The supernatant was dried by using a rotary evaporator (Buchi, Switzerland) at 45 °C to obtain the ethanolic extract (CGEtOH). The percent yield of CGEtOH was calculated by comparison to 100 g of the *C. gigantea* dry powder. CGEtOH (300 g) was dispersed in water (the ratio of CGEtOH to water was 10 g: 200 mL) and partitioned by dichloromethane (the ratio of water to dichloromethane was 200 mL:400 mL) 3 times; then, the solvent was removed to obtain the dichloromethane fraction (CGDCM). The water layer was sequentially fractionated by ethyl acetate with the same volume of dichloromethane 3 times and then combined and evaporated to obtain the ethyl acetate fraction (CGEtOAc). The remaining water layer was freeze-dried (Martin Christ, Gamma 2–16 LSC model, Germany) to obtain the water fraction (CGW). The percent yields of three fractions, CGDCM, CGEtOAc and CGW, were calculated by comparison to 100 g of CGEtOH. The extraction and fractionation protocols are illustrated in Fig. 1. All tested extracts were stored in a refrigerator (4 ± 3 °C) until use. All solvents (AR grade) were purchased from LabScan, Thailand.

#### Determination of phytochemical contents

##### Determination of cardiac glycoside

The slightly modified method described by Tofighi et al.^146^ was used to determine the total cardiac glycoside content of the *C. gigantea* stem bark extracts. Briefly, each extract (1 mg) was dissolved in 50% aqueous ethanol (1 mL) and then mixed with Baljet's reagent (1 mL), which was freshly prepared [1% picric acid (95 mL) mixed with 10% NaOH solution (5 mL)]. The mixture was allowed to stand in the dark at room temperature (30 ± 5 °C) for 1 h before being diluted with purified water (2 mL). The absorbance (482 nm) of the reaction solution was measured by using a UV/Vis spectrophotometer (Shimadzu UV-1800, Japan). The total cardiac glycoside content of each extract was calculated from the calibration curve of digoxin (Sigma–Aldrich, USA, 5–50 µg/mL, Y = 0.018X + 0.03, R^2^ = 0.9945, where Y represents the absorbance of digoxin at 482 nm, X represents the digoxin concentration (µg/mL), and R^2^ is the linear correlation coefficient. The average values ± standard deviation values (S.D.) from three independent experiments are reported in milligrams of digoxin equivalents per gram of extract (mg DXE/g extract).

##### Total triterpenoid content

The colorimetric assay using a vanillin-acetic acid reagent and sulfuric acid described by Chang et al.^147^ was used to measure the total triterpenoid content of the extracts. Briefly, the tested extracts were dissolved in glacial acetic acid (1 mg/mL 200 µL) followed by adding one mL of 5% vanillin-acetic acid solution and then 1.8 mL of sulfuric acid. The reaction mixture was heated in a water bath (70 °C) for 30 min and then cooled to ambient temperature (27 ± 2 °C). In the last step, 2 mL of glacial acetic acid was added and rigorously mixed. The absorbances (548 nm) of the reaction mixture were determined and then used to calculate the total triterpenoid contents of the extracts reported as milligrams of ursolic acid equivalents per gram of extract (mg UAE/g extract). The calibration curve of ursolic acid (Tokyo Chemical, Japan) in the range of 2 − 40 µg/mL provided a linear equation as follows: Y = 0.045X + 0.032, R^2^ = 0.9995, where Y was the absorbance value of ursolic acid (548 nm) and X was the ursolic acid concentration (µg/mL). The experiments were performed in triplicate.

##### Total phenolic content

The methods described by Baba and Malik^148^ with slight modification were used to determine the total phenolic contents of the *C. gigantea* extracts. One milliliter of sample solution in methanol (1 mg/mL) was added to 10% Folin-Ciocalteu reagent in water (Merck, Germany, 1 mL) and then rigorously mixed for 5 min. Saturated sodium bicarbonate (60 g/L, 1 mL) was added, and the reaction mixture was kept in the dark at ambient temperature (27 ± 2 °C) for 90 min. The absorbance (725 nm) values of the mixtures were measured by using a UV/Vis spectrophotometer. The calibration curve of gallic acid (1.7–13.3 µg/mL, Sigma–Aldrich, China) was used to calculate the total phenolic contents of the extracts (Y = 0.144X − 0.066, R^2^ = 0.9973, where Y represents the absorbance value of gallic acid at 725 nm, and X represents the gallic acid concentration (µg/mL). The average values of the total phenolic contents of the tested extracts ± SD (n = 3) are displayed as milligrams of gallic acid equivalents per gram of extract (mg GAE/g extract).

##### Total flavonoid content

The aluminum chloride colorimetry described by Baba and Malik^148^ and Silva et al.^149^ with some modifications was used to determine the total flavonoid contents of the extracts from *C. gigantea*. Briefly, the extract solution in 50% aqueous ethanol (1 mg/mL, 1 mL) was reacted with 2% aluminum chloride in methanol (1 mL). The reaction mixture was allowed to stand in the dark at room temperature (27 ± 2 °C) for 25 min. The absorbances (415 nm) of the reaction mixtures were determined. The results were expressed as milligrams rutin equivalents per gram of extract (mg RTE/g extract, n = 3). The calibration curve of rutin (Sigma–Aldrich, USA, 10–100 µg/mL) provided a linear equation (Y = 0.0251X + 0.0053, R^2^ = 0.9989, where Y is the absorbance value of rutin at 415 nm and X is the rutin concentration (µg/mL)) and was used to calculate the total flavonoid content of the tested samples.

##### Total alkaloid content

The total alkaloid contents of the *C. gigantea* extracts were measured using a slightly modified method described by Rajendra et al.^28^, Patel et al.^150^, and Priti and Rani^151^. Ten milligrams of the tested sample was dissolved in 1 mL of 2 N HCl and extracted with chloroform 3 times for a total volume of 10 mL. The combined chloroform layer was discarded. The acidic water layer was neutralized with NaOH (0.1 N). The bromocresol green solution (5 mL) and the pH 4.7-phosphate buffer solution (5 mL) were added and mixed well. The reaction mixture was extracted three times with chloroform (3, 3, and 4 mL). The combined chloroform layer was analyzed at 420 nm. The total alkaloid content of the extracts was calculated and expressed as milligrams berberine chloride equivalents per g extract (mg BCE/g extract, n = 3). The calibration curve of berberine chloride (Sigma Aldrich, USA, 2 − 16 µg/mL) was Y = 0.034X − 0.028, R^2^ = 0.9963 (where Y is the absorbance value of berberine chloride (420 nm) and X is the berberine chloride concentration (µg/mL)).

##### Calactin content

The content of calactin, as one of the major cardenolides found in *C. gigantea*, in *C. gigantea* stem bark extracts (CGEtOH, CGDCM, CGEtOAc and CGW) was quantified by high-performance liquid chromatography (HPLC). A calactin standard, a gift from Professor Zhi-Hong Jiang and Dr. Li-Ping Bai, Macau University of Science and Technology, Macau, was isolated and purified from the latex of *C. gigantea*, and characterized as described in a previous report^30^, and its molecular weight was reconfirmed by high-resolution mass spectrometry (C_29_H_40_O_9_, exact mass = 532.2672, calculated and found *m/z* of [M + HCOO]^-^ = 577.2654 and 577.2644, respectively, with a difference of 1.73 ppm, as shown in Fig. 3a). The mass spectrometer (an Agilent 6540 UHD Accurate-Mass quadrupole time-of-flight liquid chromatograph mass spectrometer, Agilent Technologies) equipped with a dual electrospray ionization source operated in negative mode (*m/z* range 200–800) was used to determine the accurate mass. The nebulizer pressure (N_2_) was set at 30 psi, while the drying gas flow rate was 10 L/min, and the drying gas temperature was 350 °C. The slightly modified HPLC method suggested by Kharat and Kharat^31^ was used to quantify the calactin content of the extracts. Briefly, 20 µL of the tested extracts was dissolved in methanol (5 mg/mL) and then injected into the HPLC system (Shimadzu pump LC-10ATvp, Japan) with a flow rate of 1 mL/min for 15 min using an ultraviolet/visible detector (222 nm). The stationary phase was a Phenomenex Luna® C18(2) column (150 mm × 4.6 mm, 3 µm). The mobile phase was a solution of 55% aqueous methanol (HPLC grade, LabScan, Thailand) used for isocratic elution. The content of calactin in each fraction was determined by the peaks appearing at approximately 11.43 ± 0.16 min, which corresponded to the peak of the calactin standard, as shown in Fig. 2b–f. The calactin standard curve (0.2–100 µg/mL, Y = 38682X − 16,476, R^2^ = 0.9994, where Y = peak area at a retention time of 11.43 ± 0.16 min and X = concentration of calactin (µg/mL)) was used to calculate the calactin contents. The results are expressed as mg calactin per ten grams of extract (mean ± S.D.) from three independent experiments).

### In vitro experiments

#### Cell culture

The human hepatocellular carcinoma HepG2 (JCRB1054) cell line (JCRB Cell Bank, Japan) was cultured in Dulbecco’s modified Eagle medium (DMEM) (Corning, USA) containing 10% fetal bovine serum (Gibco, USA) and 1% penicillin and streptomycin (Gibco, USA). Cells were incubated at 37 °C in a 5% CO_2_ incubator. The medium was replaced every two days. Cells were subcultured following approximately 80–90% cell confluency.

#### Cell viability detection by MTT assay

HepG2 cells were seeded in 96-well plates at a density of 1.5 × 10^4^ cells/well and incubated for 24 h. Cells were treated with various concentrations of 4 fractions of the extract from *C. gigantea*, including CGEtOH, CGDCM, CGEtOAc, and CGW. These extracts were dissolved in 1% DMSO (Sigma, USA). Doxorubicin (Adrim, Fresenius Kabi Oncology Ltd., India) was used as the positive control. After 24 h of treatment, the cells were incubated with 2 mg/mL 3-(4,5-dimethylthiazol-2-yl)-2,5-diphenyl-2H-tetrazolium bromide (MTT) solution (MERCK, Germany) and incubated at 37 °C for 4 h. The mitochondrial reductase enzyme can change MTT (yellow color) into purple formazan crystals, representing cell viability. Then, DMSO was added to dissolve this crystal, and the optical density (OD) was detected at 595 nm by a microplate reader (Synergy, BioTek, USA). The percentage of cell viability compared with the vehicle control was calculated by GraphPad Prism 9. We also explored the combination effects of *C. gigantea* extracts with DOX in further experiments using the fraction and DOX exhibiting the lowest half maximal inhibitor concentration (IC_50_) value.

#### Apoptosis assessment by immunocytochemistry

After 24 h of treatment, HepG2 cells were harvested, fixed in 4% formaldehyde solution, and stained with cleaved caspase-3 to evaluate the expression in HepG2 cells under conjugation with a fluorescence probe according to a modified protocol reported previously^152^. Cleaved caspase-3 primary antibody (AB9260, Merck, Germany) was incubated in cells that grew on glass cover slips at 4 h overnight and then incubated with goat anti-rabbit IgG H&L (Alexa Fluor® 488) (ab150077, Abcam, USA). The nucleus was incubated with Hoechst 33342. The expression was visualized under a fluorescence microscope (fluorescence adaptor), BX53F2, OLYMPUS Corporation, Japan).

#### Cell invasion assay

The cell invasion phenotype was assessed using a Boyden chamber assay^153^. The polycarbonate membranes of 8 µm pore size-transwell (3422, Costar, USA) coated with 0.4 mg/ml Matrigel (356234, Corning, USA) per 100 µL in DMEM were inserted into the upper chamber. Then, HepG2 cells at a density of 5 × 10^4^ cells seeded onto a precoated Transwell insert in the upper chamber were treated with groups of treatments in serum-free medium. DMEM supplemented with 10% FBS was added to the lower chamber. Cells were allowed to invade at 37 °C and 5% CO_2_ for 24 h. Cells in the upper chamber were removed, and the invaded cells were fixed with 4% paraformaldehyde and stained with 4% crystal violet for imaging and counting under an inverted microscope (fluorescence adaptor) (U-LH100HG (IX71), Olympus Corporation, Japan) (× 20 magnification).

#### Intracellular ATP level assay

ATP levels in cells were measured using an ATP Colorimetric Assay Kit (E-BC-K157-S, Elabscience, USA) with the principle of creatine phosphate generation from the action of creatine kinase catalyzing ATP and creatine substrates. This product was then detected by phosphomolybdic acid colorimetry. The OD was detected at 636 nm by a microplate reader. The percentage of ATP levels compared with the vehicle control was calculated by GraphPad Prism 9.

### In vivo experiments

#### Animal study

Seven-week-old male Jcl:SD rats were purchased from Nomura Siam International (Nomura Siam International Co., Ltd., Bangkok, Thailand). The animals were allowed to acclimate for 1 week after transfer with free access to water and food and were housed in a standard animal laboratory with a 12-h light–dark cycle and constant environmental conditions at the Center for Animal Research of Naresuan University (NUCAR), Naresuan University, Thailand. The approval animal ethical number is NU-AE610732. The animal procedures were approved by the Naresuan University Animal Care and Use Committee (NUACUC), Naresuan University, Thailand. In addition, the study was carried out in compliance with the Animal Research: Reporting In Vivo Experiments (ARRIVE) guidelines. All of the requirements specified in the animal protocols, as well as the data collected, were assembled using the ARRIVE Essential 10 guidelines.

Rats were intraperitoneally injected (IP) with N-nitrosodiethylamine (DEN) (CAS 55-18-5, TCI, Japan) at a dose of 50 mg/kg body weight (BW) twice a week for 3 weeks and then injected with a dose of 30 mg/kg BW twice a week for another 5 weeks until week 8 as reported by Yong-fanf Ding et al. and Gabriela Velasco-Loyden et al. with some modifications^49,92^. After stopping DEN injection, the rats were left untreated for 4 weeks (week 8 to week 12), which allowed for HCC development. The body weight of the animals was measured before performing each treatment throughout the study.

The experimental design is illustrated in Fig. 6. At week 12, the animals were divided into five groups and treated for 4 weeks, with three IP injections per week: Group (1) was the vehicle control, Group (2) was IP injection with DOX at 0.5 mg/kg, Group (3) was IP injection with CGDCM at 2.5 mg/kg (CGDCM-L), Group (4) was IP injection with CGDCM at 5 mg/kg (CGDCM-H), and Group (5) was IP injected with DOX 0.5 mg/kg + CGDCM-H at 5 mg/kg.

At week 16, all rats were finally euthanized with 50 mg/kg thiopental sodium IP injection. Blood was collected via the abdominal aorta, and internal organs, including the liver, heart, lungs, intestines, kidneys, testis, and femur bone, were removed for tissue histological analysis. The liver was weighed and photographed after washing with ice-cold saline solution. All of these organs were fixed in 10% neutral buffered formalin after being washed with ice-cold saline solution.

#### Determination of serum AST and ALT

At weeks 0, 3, 8, and 12, blood was taken from the tail vein, and at week 16, blood was taken from the abdominal aorta. Serum liver damage markers, alanine aminotransferase (ALT) and aspartate transaminase (AST)^154,155^ were measured by the Biomedical Laboratory, Phitsanulok, Thailand.

#### Histopathological, immunohistochemical and immunofluorescence evaluation

##### Hematoxylin and eosin (H&E) staining

The organs were fixed in 10% neutral buffered formalin and embedded in paraffin. Formalin-fixed and paraffin-embedded tissue slices of 3 to 5 µm were produced. Deparaffinized tissue sections were stained with hematoxylin and eosin (C.V. Laboratories, Thailand) for the histological study. Evaluation of liver morphology was initially performed with hematoxylin and eosin-stained (H&E) sections as well as other organs, including the heart, lung, kidney, small and large intestines, testes, and bone marrow of the femur, by using an Olympus BX53 microscope with a DP26 digital camera (Olympus, Japan). Based on H&E sections, hepatocellular carcinoma (HCC) was diagnosed in the presence of enlarged neoplastic hepatocytes with a high N/C ratio, nuclear pleomorphism, and eosinophilic cytoplasm that arrange in a thick trabecular or solid pattern and absence of a lobular pattern in the liver mass^156^.

##### Masson’s trichrome staining

For evaluation of liver fibrosis, deparaffinized 3- to 5-µm liver tissue sections were subjected to Masson’s trichrome staining using a Masson–Goldner staining kit (Merck, Germany) according to the manufacturer’s protocol. Weigert’s iron hematoxylin stains the nuclei in dark brown to black, while components such as muscle, cytoplasm, and erythrocytes are stained with azophloxin and orange G solution in red or orange. The connective tissue appears green by the light green SF solution counterstain. Liver fibrosis is diagnosed by the presence of connective tissue in the liver above the normal low rate seen in portal areas. Prominent or severely increased fibrosis surrounding hepatic lobules and bridging between adjacent portal areas with or without regenerative response is considered to represent cirrhosis^156^.

##### Immunohistochemistry

The immunohistochemical study was performed in deparaffinized 3- to 5-µm tissue sections. For evaluation of cell proliferation, Ki-67 antigen in liver lesions was determined by anti-Ki-67 polyclonal antibody (1:100, AB9260, Merck, Germany). Detection was performed using a goat anti-mouse HRP secondary antibody (A28177, Invitrogen, USA) at a dilution of 1:100 followed by colorimetric detection using 3,3'-diaminobenzidine (DAB). Finally, tissues were counterstained with hematoxylin. Immunoreactivity was observed in the nucleus. The intensity and percentage of nuclei with positive staining (brown color) in cancer cells and noncancerous liver cells were evaluated and compared.

##### Apoptosis detection by immunofluorescence

Apoptosis was investigated by cleaved caspase-3 expression in a 3 µm thick paraffin-embedded liver tissue section. Hoechst staining was used to distinguish condensed pyknotic nuclei in apoptotic cells. Liver tissue slides were exposed to sodium citrate buffer at 45 °C for 45 min and were then allowed to cool for 20 min. The slides were then incubated for 15 min with 3% H_2_O_2_, followed by 40 min of nonspecific blocking with 4% FBS in 1 × PBS. The primary antibody against cleaved caspase-3 (AB9260, Merck, Germany) was incubated in a liver slide at 4 °C for 4 h. After that, a slide was incubated for 2 h with goat anti-rabbit IgG H&L (Alexa Fluor 488) (ab150077, Abcam, USA). Then, Hoechst 33342, a nucleic acid dye, was applied to the slide. Finally, images of the fluorescence signal were visualized and captured with a fluorescence microscope (BX53F2, Olympus Corporation, Japan).

#### Liver protein expression assay by western blotting

Protein levels in liver tissues were extracted using RIPA buffer (150 mM NaCl, 50 mM Tris–HCl pH 7.5, 0.1% SDS, 1% Triton X-100, 0.5% sodium deoxycholate, and 1 mM EDTA)^157^ containing proteinase inhibitor cocktail (ML051, HIMEDIA, India). Cell lysates were collected, and the concentration was quantified by bicinchoninic acid assay reagent (Thermo Fisher Scientific, USA). By electrophoresis on a sodium dodecyl sulfate polyacrylamide gel, equal amounts of proteins were separated and transferred to a polyvinylidene fluoride membrane. Then, the membranes were incubated with OneStep Blocker solution (BS001, Genedirex, USA), followed by anti-cleaved caspase-3 (Thermo Fisher Scientific, USA), IL-6 (Abcam, USA), TNF-α (Merck, Germany), TNF-β1 (Abcam, USA), and α-SMA (Cell Signaling, USA) primary antibodies and then exposed to horseradish peroxidase-conjugated goat anti-rabbit secondary antibody (Cell Signaling, USA) or goat anti-mouse secondary antibody (Invitrogen, USA). β-actin (Cell Signaling, USA) was used as an internal control. Finally, protein bands were visualized using Luminata TM Forte Western HRP Substrate (Merck, Germany) and detected by chemiluminescence western blot detection (Image Quant LAS 4000; GE Healthcare Life Science, USA). Percentages of relative expression levels of protein/actin were calculated by ImageJ software version 1.46.

#### Liver fatty acid levels

The Free Fatty Acid Quantification Assay kit was used to determine the amounts of free fatty acids (ab65341, Abcam, US). Ten milligrams of liver tissue was homogenized in a chloroform-Triton-X 100 solution (1% Triton X 100 in pure chloroform). To eliminate chloroform from the samples, the solution was centrifuged and air-dried. Fatty acid assay buffer was used to dissolve the dried lipid, and then acyl-CoA synthase was added. The enhancer and the fluorescent probe were added. A fluorescence microplate reader detected the fluorescence signal at Ex/Em 530/590 nm.

#### Liver ATP levels

ATP levels in liver tissues were measured using an ATP Colorimetric Assay Kit (E-BC-K157-S, Elabscience, USA) according to the manufacturer’s instructions. Briefly, 10 mg of liver tissue was homogenized in boiling double distilled water and centrifuged at 3500 rpm for 10 min, and the supernatant was collected to measure creatine phosphate production from creatine kinase catalyzing adenosine triphosphate, which was then detected by phosphomolybdic acid by colorimetry at an OD of 636 nm using a microplate reader.

#### Statistical analysis

Data from three independent experiments are expressed as the mean ± SD using one-way analysis of variance (ANOVA) or Student's *t test* with Tukey's post-hoc analysis to determine the statistical significance of differences between the experimental and control groups by Graph Prism Software version 9.
